# Prolonged transition time between colostrum and mature milk in a bear, the giant panda, *Ailuropoda melanoleuca*

**DOI:** 10.1098/rsos.150395

**Published:** 2015-10-21

**Authors:** Kate Griffiths, Rong Hou, Hairui Wang, Zhihe Zhang, Liang Zhang, Tong Zhang, David G. Watson, Richard J. S. Burchmore, I. Kati Loeffler, Malcolm W. Kennedy

**Affiliations:** 1 Institute of Biodiversity, Animal Health and Comparative Medicine, College of Medical, Veterinary and Life Sciences, University of Glasgow, Graham err Building, Glasgow, G12 8QQ, UK; 2 The Sichuan Key Laboratory for Conservation Biology on Endangered Wildlife, Chengdu Research Base of Giant Panda Breeding, 1375 Panda Road, Northern Suburb, Chengdu, Sichuan Province 610081, People’s Republic of China; 3 Strathclyde Institute of Pharmacy and Biomedical Sciences, University of Strathclyde, 161 Cathedral Street, Glasgow, G4 0RE, UK; 4 Institute of Infection, Immunity and Inflammation and Glasgow Polyomics, College of Medical, Veterinary and Life Sciences, University of Glasgow, Garscube Campus, Glasgow, G12 1QH, UK

**Keywords:** giant panda, *Ailuropoda melanoleuca*, milk, colostrum, proteins, oligosaccharides

## Abstract

Bears produce the most altricial neonates of any placental mammal. We hypothesized that the transition from colostrum to mature milk in bears reflects a temporal and biochemical adaptation for altricial development and immune protection. Comparison of bear milks with milks of other eutherians yielded distinctive protein profiles. Proteomic and metabolomic analysis of serial milk samples collected from six giant pandas showed a prolonged transition from colostrum to main-phase lactation over approximately 30 days. Particularly striking are the persistence or sequential appearance of adaptive and innate immune factors. The endurance of immunoglobulin G suggests an unusual duration of trans-intestinal absorption of maternal antibodies, and is potentially relevant to the underdeveloped lymphoid system of giant panda neonates. Levels of certain milk oligosaccharides known to exert anti-microbial activities and/or that are conducive to the development of neonatal gut microbiomes underwent an almost complete changeover around days 20–30 postpartum, coincident with the maturation of the protein profile. A potential metabolic marker of starvation was detected, the prominence of which may reflect the natural postpartum period of anorexia in giant panda mothers. Early lactation in giant pandas, and possibly in other ursids, appears to be adapted for the unique requirements of unusually altricial eutherian neonates.

## 1. Introduction

The composition of mammalian milks and the duration of lactation vary dramatically from species to species, influenced by the ecology, evolutionary history, placental type and the developmental state of the neonate at birth [1–3]. Marsupials have extremely altricial neonates and much longer lactation periods than do eutherians of similar body size and ecology, indicative of an extended period of dependence on maternally derived nutrition and immunological support after birth [4]. Even among eutherians that live in similar environments, there are dramatic differences in lactation periods that reflect diverse feeding and reproductive ecologies. Compare, for example, the short lactation periods in Phocidae (4 days in hooded seals) to the prolonged periods in Otariidae (4–24 months in fur seals and sea lions) and Odobenidae (1–2 years in walruses) [5–7].

Marsupials exhibit dramatic changes in milk composition with time after birth to nurture what is essentially an extra-uterine gestation [4,8]. For example, certain milk proteins are only present early in lactation and then disappear, to be replaced by others that disappear and are replaced in turn through the course of lactation [4,9,10]. Such dramatic and sustained changes in milk proteins do not occur in eutherian mammals, presumably because their neonates are at a significantly more advanced stage of development at birth. Eutherian milks do, however, change radically in the immediate period after birth. The composition of milk during this colostrum phase reflects the type of placenta characteristic of the species [1].

Eutherians with haemochorial placentae (e.g. many primates and rodents) commonly transfer immunoglobulins transplacentally such that the neonate is born with a sample of maternal antibodies equivalent to the mother’s own plasma levels [11,12]. In species possessing endotheliochorial placentae, trans-placental immunoglobulin transfer is rare, and the neonate must obtain immunoglobulins via colostrum immediately after birth for direct transfer into the circulation [1,13]. For this, junctions between intestinal cells are open for only about 6–24 h postpartum [13]. Timing of the closure of intercellular junctions in the gut (‘gut closure’) has been examined in only a few species. Whether a similar interval of trans-intestinal immunoglobulin transfer also applies to ursids—the subject of this paper—is not known.

Among eutherian mammals, the neonates of bears are altricial to an unusual degree, with the lowest ratio of neonatal to maternal body mass, despite small litter sizes [14]. Among bears, neonates of the giant panda, *Ailuropoda melanoleuca*, are the most altricial, with a neonatal to maternal body mass ratio of about 1:1000. They are born deaf, blind and virtually naked of hair (see electronic supplementary material, figure S1). Moreover, their lymphoid system is particularly underdeveloped, emphasizing a potentially unusual degree of dependency on maternal immunity among eutherians [15]. We know of no published description of the type of placenta exhibited by giant pandas, but those ursids for which there is information have endotheliochorial discoidal zonary placentae [16]. If this is also true of giant pandas, then their cubs may be particularly dependent on colostrum for immunity during the neonatal period.

Colostrum, compared with mature milk, is generally richer in protein than lipids and carbohydrates, particularly in species in which immunoglobulin transfer is postnatal [1]. The change in concentrations of these nutrient classes between colostrum and mature milk has been examined in a range of species [1], but seldom has the time taken for the transition from colostrum to mature milk been examined in detail. Precise criteria with which to define the end of the colostral period also have not been developed. Nevertheless, species with similar body masses to bears but which produce precocious neonates appear to have short colostral periods (e.g. bovids and camels [17–19]). We here take the colostrum period to end when all the major components of milk reach an approximate steady state, although slight modifications in composition may still occur during the main phase. Given what is known about the reproductive physiology of the species for which the transition period has been described, and given the altriciality of neonatal ursids, we hypothesized that the transition period would be comparatively long in bears. Once information of the kind we present here for giant pandas becomes available for a broad sample of eutherians, then this can be fully tested in, for instance, a meta-analysis of the transition times relative to total lactation durations for species that give birth to either altricial or precocial young.

Previous investigations of giant panda milk have focused on basic analysis of major nutrients or on oligosaccharide profiling from single milk samples [20–25]. Our study examined serial milk samples from six individuals, beginning 12 h after parturition until five months into lactation, to explore how the relationship between the dynamics of milk composition might meet the developmental needs of an altricial neonate. We followed proteins and small molecules to elucidate how the components of colostrum changed with time after birth, how long the transition to a mature milk profile takes, and how such changes may align with what might be expected for a species with highly altricial young.

Our choice of the giant panda was because this bear represents an extreme in altriciality within the Ursidae. Changes in proteins and small anti-microbial defence molecules were followed with time after birth, and a prolonged transition time between colostrum and mature milk lasting about 30 days was apparent. Some of the most remarkable changes during that period were in the dynamics of proteins and oligosaccharides involved in antibacterial defence or in the establishment of an appropriate neonatal gut microbiome (‘probiotic’ [26,27]). The latter may be particularly relevant to giant pandas in their progression from a milk-based to a predominantly vegetarian diet despite an anatomically carnivorous digestive system [28,29].

## 2. Material and methods

### 2.1 Milk collection and processing

All giant panda samples were collected from captive-bred animals at the Chengdu Research Base of Giant Panda Breeding, Chengdu, Sichuan Province, People’s Republic of China, during the years 2006, 2007, 2011 and 2012. Six animals were sampled in all, two of which were sampled over multiple lactations. In this facility, cubs are routinely removed from their mothers at three to six months of age to induce the females to enter oestrus again for the next season. Lactation and nursing in giant pandas is usually taken to last for about nine months, although the average duration in the wild is not known and captive-reared cubs have been observed to suckle for as long as two and a half years. A complete list of sampling dates, individual animal names and studbook numbers is given in the electronic supplementary material, table S1. The health status of each animal was monitored regularly. While overt disease was not observed in any donor, minor ailments and suboptimal blood factor levels were observed in some. Mother pandas normally enter a period of anorexia for 7–14 days postpartum, but the animals from whom samples were obtained were given glucose before normal diet resumed.

Sample times for other species were all from main-phase lactation as follows: bottlenose dolphin, *Tursiops truncatus*, one animal sampled 9 months after birth (average lactation period for the species 18–20 months); Indian elephant, *Elephas maximus indicus*, one animal sampled 2 months after birth (average lactation period 2–6 years); African bush elephant, *Loxodonta africana*, one animal sampled 15 months after birth (average lactation period about 2 years); polar bear, *Ursus maritimus*, one animal sampled opportunistically after emergence from winter den, estimated 3–4 months after birth (average lactation period 24–28 months); grizzly bear *Ursus arctos horribilis*, one animal sampled at estimated 3–4 months after birth (average lactation period 1.5–3 years). Human milk was from an anonymous donor in Scotland, mid-lactation; cow, *Bos taurus*, pooled commercial milk sample (average lactation period 9–10 months); dog, *Canis familiaris*, one animal sampled at mid-lactation (average lactation period 8 weeks).

All non-domesticated species except the polar bear were captive-bred, and milk samples were obtained by their keepers. Milk samples from all these animals except the polar and grizzly bears were obtained by hand from food-rewarded, conscious animals who were not anaesthetized, sedated, drug-treated or physically restrained. Milk from polar bears was obtained opportunistically from anaesthetized, wild animals during fieldwork in Svalbard, Norwegian Polar Institute. Grizzly bear milk was sampled opportunistically from anaesthetized, captive bears treated with oxytocin to facilitate milk let-down at the research facility of Washington State University, Pullman, WA, USA. In all the cases, samples were collected from a single mammary gland and were not pooled between individuals or over time. Sample sizes ranged from 0.5 to 5 ml, depending on the species and quality of milk let-down. The mammary glands were never emptied, as sampling was not to interfere with the nutrition available to the offspring.

Giant panda milk samples were stored either in liquid nitrogen or at −80°C immediately after collection, transferred to Glasgow frozen, and stored at −20°C until use. Samples from other species were frozen immediately after collection and stored at −20°C. Milks were centrifuged at 4°C for 10 minutes at 3000*g* in a bench-top centrifuge or at 12 000*g* in a microfuge. The buoyant fat layer and pellet were removed, and the intermediate, aqueous phase fluid layer collected and used immediately or stored at −20°C until use.

### 2.2 Protein electrophoresis

One-dimensional (1D) vertical sodium dodecyl sulfate polyacrylamide gel electrophoresis (SDS-PAGE) was carried out using the Invitrogen (Thermo Scientific, Paisley, UK) NuPAGE SDS-PAGE system with precast 4–12% gradient gels, following the manufacturer’s instructions. Gels were stained for protein using Coomassie Blue or InstantBlue (Expedion, Harston, UK). *β*2-Mercaptoethanol (25 μl added to 1 ml sample buffer) was used as reducing agent for reducing conditions. Images of gels were recorded using a Kodak imager. Electronic images were not modified other than for contrast and brightness adjustments. Pre-stained molecular mass/relative mobility (*M*_r_) standard proteins were obtained from New England Biolabs, Ipswich, MA, USA (cat. number P7708S). Preparative two-dimensional (2D; isoelectric focusing on a pH 4–7 immobilized pH gradient in the first dimension, SDS-PAGE on a 10% homogeneous gel in the second) gels were run using an Ettan DALT electrophoresis system (GE Healthcare, Little Chalfont Buckinghamshire UK) as previously described [30], except that gels were loaded with 30 μl milk sample processed as described above. Gels were stained with Coomassie Blue. These gels provided sufficient protein from excised gel spots for identification by proteomics procedures. 2D difference gel electrophoresis (DIGE) was carried out as previously described [30]. Five microlitres of milk sample from day 1 postpartum were labelled with Cy3, and 5 μl of milk sample from day 152 postpartum were labelled with Cy5, prior to mixing and electrophoresis.

### 2.3 Proteomics

Stained protein bands or spots were excised from preparative 1D or 2D gels, respectively, and analysed by liquid chromatography–mass spectrometry (LC-MS) as previously described [31]. Protein identifications were assigned using the MASCOT search engine to interrogate protein and gene sequences in the NCBI databases. Searches were restricted to the giant panda resources specifically, or to dog sequence databases where panda gene annotations were still incomplete, and allowed a mass tolerance of 0.4 Da for both single and dual mass spectrometry analyses.

### 2.4 Metabolomics

LC-MS was carried out by using an Orbitrap Exactive LC-MS system. The electrospray ionization interface was operated in a positive/negative polarity switching mode. The spray voltage was 4.5 kV for positive mode and 4.0 kV for negative mode. The temperature of the ion transfer capillary was 275°C; sheath and auxiliary gas were 50 and 17 arb. units, respectively. The full scan range was 75–1200 *m*/*z* for both positive and negative modes with settings of automatic gain control target and resolution as Balanced and High (1×10^6^ and 50 000), respectively. The data were recorded using Xcalibur v. 2.1.0 software package (Thermo Fisher Scientific). Mass calibration was performed for both electrospray ionization polarities before the analysis, using the standard Thermo Calmix solution with inclusion of some additional compounds to cover the low mass range. The signals of 83.0604 *m*/*z* (2× acetonitrile-H) and 91.0037 *m*/*z* (2× formate-H) were selected as lock masses for positive and negative mode, respectively, during each analytical run. The mobile phases used in hydrophilic interaction liquid chromatography conditions were 20 mM ammonium carbonate buffer (pH 9.2; A) and acetonitrile (B) (ZIC-pHILIC column (150×4.6 mm, 5 μm); Merck Millipore, Darmstadt, Germany). The gradient programme was as follows: B 80% (0 min); B 20% (30 min); B 8% (31 min); B 8% (36 min); B 80% (37 min); B 80% (46 min). The flow rate was 0.3 ml min^−1^. Peaks were extracted by using Sieve software (Thermo Fisher Scientific) and, using a macro written in Microsoft Excel, matched against the human metabolome database. Putative identification of compounds was based on elemental compositions within 3 ppm of the exact mass, which excludes alternative compositions but does not exclude isomers. Semi-targeted profiling for oligosaccharides was performed by manually searching accurate masses (±3 ppm) reported in the published bear milk studies or predicted based on the combination of five basic building blocks for mammalian milk oligosaccharides.

## 3. Results

### 3.1 Comparison of giant panda milk protein profiles with milks of other mammals

**Figure 1.**
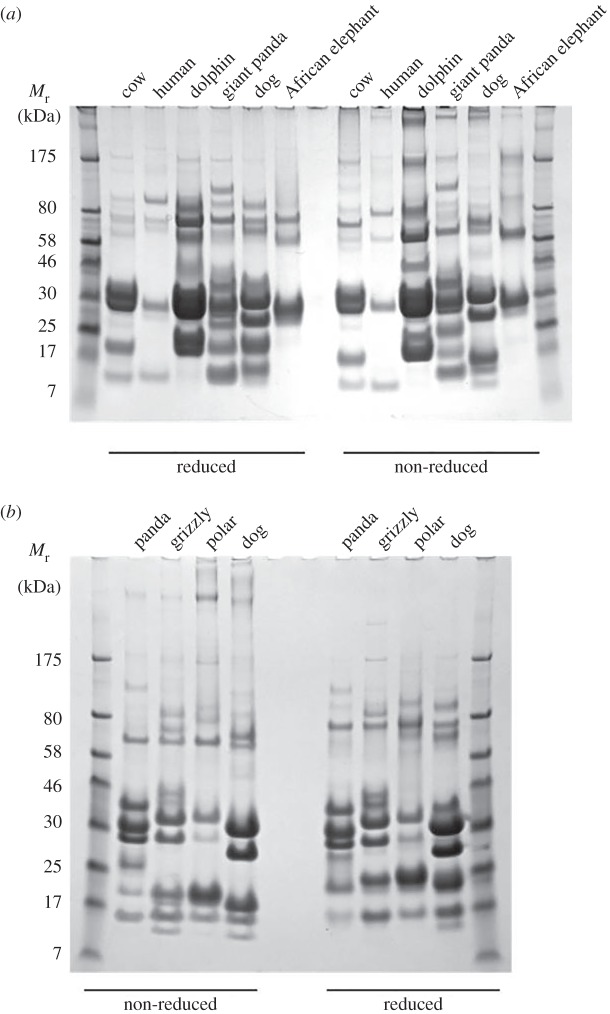
Giant panda milk proteins compared with those of other mammals. SDS-PAGE analysis of milks from several species of mammal (*a*). African elephant milk is closely similar in protein profile to that of Indian elephants (not shown). A similar analysis of the milks from three species of bear, and another member of the Carnivora, the domestic dog, is given in (*b*). The giant panda sample was from Shu Qing, postpartum day 46. All samples were collected during the main phase of lactation, as described in Material and Methods. The gels were run under reducing and non-reducing conditions as indicated. Relative mobilities (*M*_r_) of protein size standards are as indicated in kiloDaltons (kDa).

We first investigated how similar or different ursid milks are to milks of other species. Milks collected during mid-lactation from several species were analysed by 1D protein gel electrophoresis (figure 1*a*), which highlighted the considerable differences in protein profiles among species. Those from the same clade of the Carnivora (dog and giant panda [32]) or of the Cetartiodactyla (cow and dolphin [33]) show some similarities in protein band presences and absences, albeit with differences in the preponderance of various proteins. All are distinct from elephants (both African and Indian). Within the Ursidae (figure 1*b*), polar and grizzly bear milks are more similar to each other than to giant pandas, which is consistent with the close phylogenetic relationship of the former two [34–36].

### 3.2 Transition from colostrum to mature milk: proteins

**Figure 2.**
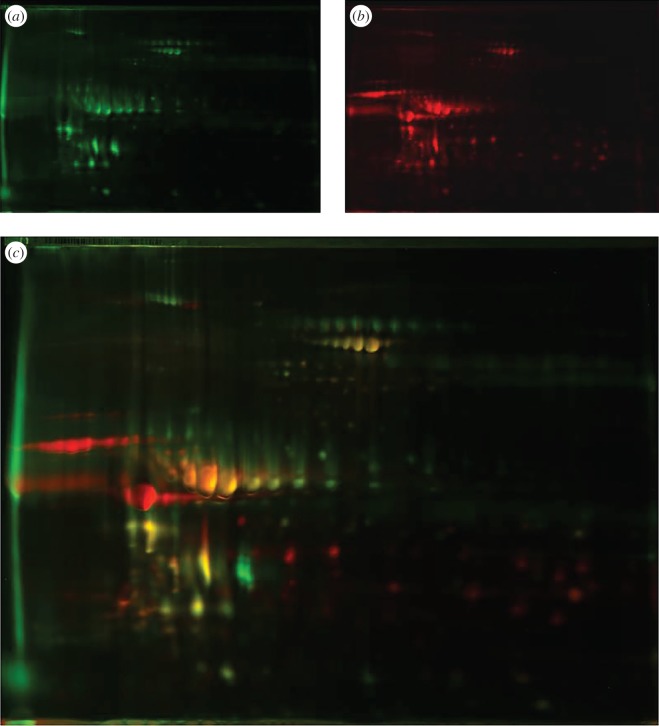
Transition of giant panda milk from colostrum to mature milk. Two-dimensional fluorescent difference gel electrophoresis (DIGE) of samples from day 1 (*a*, green) and day 152 (*b*, red) postpartum. A superimposition of the two images (*c*) indicates in yellow where the same species of protein appears in both samples. Preparative 2D gels of the same samples are shown in the electronic supplementary material, figure S2, providing protein identifications given in the electronic supplementary material, table S2.

Samples of milk taken serially from several giant pandas exhibited progressive changes in protein profile between colostrum and mature milk. 2D DIGE analysis shown in figure 2 compares samples collected on days 1 (*a*, green) and 152 (*b*, red) postpartum and analysed in the same gel. In the superimposed image (*c*), yellow indicates where protein species are present in both samples, revealing proteins disappearing or appearing, and those persisting across the transition and into the main lactation period. The loading of this type of analytical gel is inadequate for protein identification, so preparative 2D gels of the same samples were run (electronic supplementary material, figure S2), leading to protein identifications given in the electronic supplementary material, table S2.

**Figure 3.**
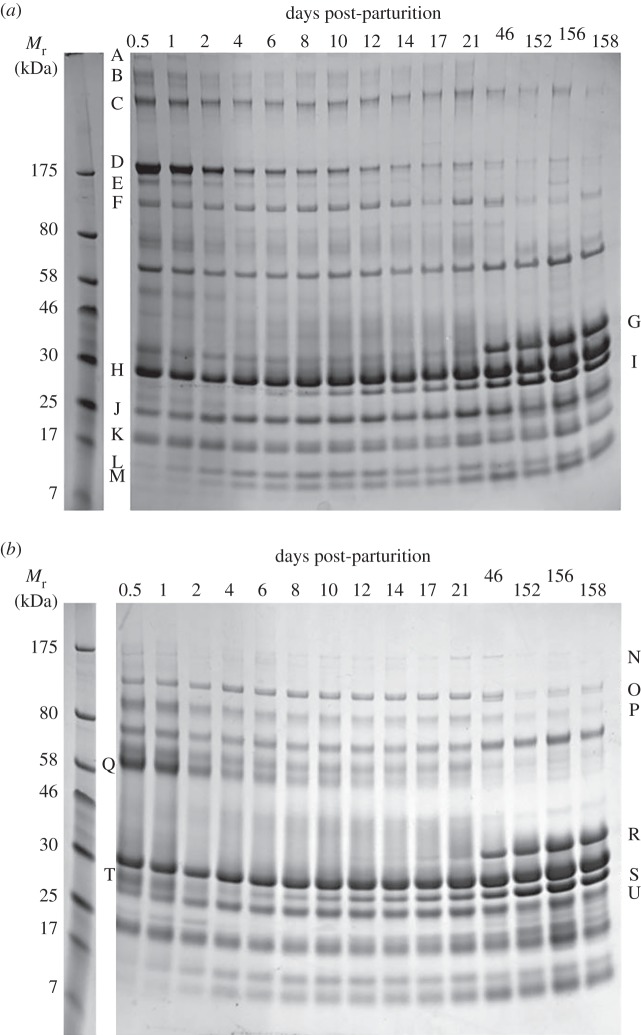
Progressive changes in protein profiles of giant panda milk from colostrum to mature milk. 1D protein electrophoresis gels loaded with (*a*) non-reduced samples and (*b*) reduced samples. Samples from days 0.5 to 21 are from Li Li; days 46 and 158, Shu Qing; day 152, Ya Ya; day 156, Qi Zhen. The protein bands indicated by letters were excised from gels and subjected to proteomic identification. These data, together with data from additional 1D and 2D gels, provided consensus identifications given in table 1 and electronic supplementary material, table S3. See also the electronic supplementary material, figure S2, with table S2 used for additional confirmation.

The time course of changes is best illustrated in 1D protein electrophoresis gels (figure 3), using samples taken periodically from 12 h after birth up to 158 days of lactation. Following the initial colostrum phase, a slow acquisition of the full mature milk protein profile is evident, along with the gradual disappearance of other protein types. Similar protein electropohoresis analysis showed that the transition in protein composition appears to be complete by 30 days (not shown). Identification of the main proteins present was carried out by combining information obtained by excision of protein bands from 1D and 2D gels (including those in figures 3 and S2) and as visualized in figure 2. The identifications from gel bands annotated in figure 3 are summarized in table 1, with additional detail given in the electronic supplementary material, table S3.

**Table 1.** Proteins in giant panda milk. Identification of proteins isolated from bands excised from the SDS-PAGE gels shown in figure 3.

protein identification^a^	found in gel band^b^	putative function and comments^c^
immunoglobulin *μ* heavy chain	A	IgM. Antibody. Abundant in serum and colostra, less so in secretions
immunoglobulin *α* heavy chain	B, C	IgA. Secretory antibody. Abundant in secretions (e.g. tears, saliva, bile, milks)
immunoglobulin λ light chain	A, T, S	light chain isoform associated with all immunoglobulin subclasses
polymeric immunoglobulin receptor	C, P	receptor for IgA and IgM mediating secretion, part of which (secretory component) remains bound to IgA to protect it against proteolytic cleavage in intestine
immunoglobulin *γ* heavy chain	D, E, Q	IgG. Antibody. Most abundant class in plasma, much less so in secretions
bile salt-activated lipase	F, O	also termed bile-stimulated or -dependent lipase. Presumed to assist with digestion of triglyceride lipids
*κ*-casein	G	stabilizes micelle formation, prevents casein precipitation
*β*-casein	H, R, S	source of amino acids, delivers calcium, phosphate, lipids, structural component of casein micelles
*β*-lactoglobulin isoform 1	G, J, K, U	binds and probably transports retinol (vitamin A), vitamin D, and fatty acids including polyunsaturated fatty acids
*β*-lactoglobulin isoform 2	G	similar to isoform 1
lactotransferrin	I	iron binding transport protein with antibacterial properties
anti-leukoproteinase	K	proteinase inhibitor
lysozyme C	L	antibacterial. Milk isoform
whey acidic protein	M	function unclear. Possibly associated with innate immunity. Possibly plays role in regulation of the proliferation of mammary epithelial cells
xanthine dehydrogenase	N	involved in milk fat globule secretion and also innate immunity
apolipoprotein D isoform 2	S	lipid transporter

^a^Gel bands as indicated in figure 3.

^b^See the electronic supplementary material, table S3 for complete listing of identifications, NCBI GenBank accession codes and MASCOT peptide search scores.

^c^The putative functions and comments are drawn from a variety of sources including NCBI and UniProtKB/Swiss-Prot databases.

Notable among the proteins present early in lactation are immunoglobulins, accompanied by the polymeric immunoglobulin receptor that is involved in secretion of IgA molecules (and IgM in some but not all species [37]). The receptor appears in the milk as the secretory component that protects IgA in particular from digestion in the intestine. IgG is not similarly protected, but instead may be absorbed into the circulation of the neonate. Among the proteins that accumulate slowly in panda milk are the caseins, which are milk-specific proteins that usually occur in high abundance in milks of other species [38,39].

### 3.3 Transition from colostrum to mature milk: small molecules

Serially sampled milks from giant pandas were also analysed for small organic compounds, revealing substantial relative changes with time in several constituents of potential importance. The compounds exhibiting the greatest peak heights and areas from mass spectrometry at three different sampling times are given in the electronic supplementary material, table S4, and are compared with human and bovine milks. Notable changes in ranking are evident with lactose (descending with time), sialyllactoses (descending) and fucosyllactose (ascending), and, in contrast with human and bovine milks, the persistence of taurine.

**Figure 4.**
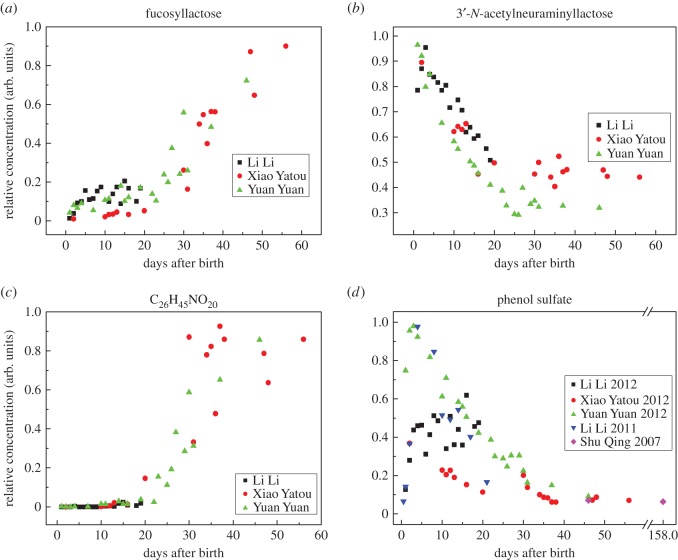
Changes in giant panda milk oligosaccharides and other small molecules with time after birth. Selection of four compounds illustrating distinctive patterns of change during the transition period. (*a*,*b*) Two oligosaccharides that show opposite trends in relative concentrations with time, the cross-over point being between 20 and 30 days postpartum. Both of these are known to have effects on microbial colonization of the gut in some species (see text). (*c*) Complete absence of an oligosaccharide that then appears abruptly from day 20. This, C_26_H_45_NO_20_, is GalNAc(*α*1–3)[Fuc(*α*1–2)]Gal(*β*1–4)Glc (a tetrasaccharide), which is the human blood group A determinant or an isomer of it. (*d*) Extremely rapid post-parturient rise, then fall, in phenol sulfate levels that may be due to the anorexic period that panda mothers endure for 7–10 days post-parturition. This graph includes data for panda Li Li taken in an earlier year (2011), as well as from an additional panda, Shu Qing, that was sampled on day 158 and confirms the eventual disappearance of phenol sulfate.

In a more detailed analysis, we found dramatic changes with time for oligosaccharides that may be present for their anti-microbial or probiotic activities rather than as a source of energy [27,40–43]. Three examples are illustrated in figure 4. Fucosyllactose was initially close to zero in concentration and then rose dramatically after 20 days (figure 4*a*), whereas 3′-*N*-acetylneuraminyllactose fell steadily with time (figure 4*b*), changeover occurring between days 20 and 30. Curiously, the 6′-*N*-acetylneuraminyllactose isomer of the latter oligosaccharide exhibited the opposite time course (electronic supplementary material, figure S3). Particularly dramatic were changes in those oligosaccharides that were undetectable until about day 20 and then rose dramatically (figure 4*c* and electronic supplementary material, figure S3). A list of these and other oligosaccharides is given in the electronic supplementary material, table S5, and their postpartum trends are illustrated in the electronic supplementary material, figure S3, which also illustrates the fall in lactose until about day 25.

Phenol sulfate was an unexpectedly abundant compound in the milk, levels of which rose very rapidly postpartum and then fell steadily to a plateau approaching zero after day 30 (figure 4*d*). Possible reasons for the unexpected presence and loss of this molecule in milk are discussed below.

## 4. Discussion

The neonates of bears are remarkable among placental mammals for their premature condition and uniquely low body mass relative to that of their mothers. Exploiting giant pandas as representing an extreme test case even among bears, we hypothesized that maturation of colostrum to mature milk would be prolonged compared with other eutherians. We indeed found a slow transition in the types of proteins and micronutrients present in giant panda milk over time. Comparative detail is available for few other species, but our findings suggest that early lactation in bears may be modified to support the development of unusually immature neonates, albeit not to the extreme of marsupials to whom the term ‘external gestation’ has been applied [8].

### 4.1 Proteins

The proteins observed to be abundant initially and then to diminish with time after parturition were the immunoglobulins. The rate at which this occurred differed among classes, IgA being the most persistent, which reflects its protective role in secretions in general, and in milks continuously throughout lactation. IgA presumably plays a protective role for both the infant and the mammary gland itself, as well as to control the neonate’s gut microbiome [44,45]. IgA is protected against proteolytic cleavage by a secretory component, which is a fragment of the polymeric immunoglobulin receptor [37], hence the latter’s persistence along with IgA. IgM and IgG probably have lesser roles in the neonatal gut because they are not as resilient as IgA to proteolytic cleavage. These two immunoglobulins are particularly prominent components of the colostra of mammals with placentae that do not permit transfer of immunoglobulins, as they must cross the intestinal epithelial barrier into the neonatal circulation before epithelial closure within hours after birth [46,47]. The persistence of IgG in giant panda colostrum is unusual in comparison to other species [17–19]. This may indicate that trans-intestinal transfer of immunoglobulins lasts for longer than the 24 h usually taken to be the maximum in other species groups [13]. Alternatively, giant panda neonates may have a mechanism for transferring IgG across the intestinal epithelia continuously, such as with the FcRn transporter which is active in IgG translocation across the intestinal epithelia in neonatal rodents but not humans [11]. The kinetics of this transition may be unique or at least highly unusual among eutherian mammals, although there is currently insufficient coverage of mammalian lineages to be sure.

Several protein types appear with time, including two isoforms of *β*-lactoglobulin. The biological functions of *β*-lactoglobulins are not entirely understood, though they are presumed to transfer essential lipids such as retinol (vitamin A), cholecalciferol (vitamin D) and (poly)unsaturated fatty acids [48,49]. *β*-lactoglobulin is a major constituent of the milks of many mammals, notable exceptions being rodents, elephants, lagomorphs and primates [49]. Most prominent among the proteins that increase in concentration are apolipoprotein D (also a lipid transporter) and the caseins. Caseins appear in all mammalian milks and have a variety of functions. In addition to their role as a source of amino acids, they serve as carriers for otherwise poorly soluble calcium phosphate to form a complex that prevents ectopic calcification of the mammary gland [38,50–52]. Furthermore, cleavage of caseins by gut proteinases induces curdling of milk to a semi-solid form [38]. Disorders of curd formation cause critical gastrointestinal disturbances in bear cubs fed unsuitable milk formulae [53,54].

Bile salt-activated lipase is persistent and seen gradually to decrease late in our sampling period. This enzyme is commonly found in milks and is thought to assist the neonate in digestion of milk triglycerides [55,56]. It is also produced in the pancreas, but probably only at low levels in neonates relative to the adult pancreas and mammary gland [57]. Its role in giant panda milk could, therefore, be to supplement a cub’s digestive processes before its own pancreatic production of the enzyme becomes adequate. It is a highly *O*-glycosylated enzyme, hence its slow mobility in protein gels (the core polypeptide in humans is 77 kDa in mass, yet its effective mass can be considerably greater [55]).

Haptoglobin was found in samples taken very early in lactation (electronic supplementary material, figure S2 and table S2). This is a classic acute phase protein produced by the liver during acute phase (fever) responses. Its protective functions include bacteriostasis, scavenging free haemoglobin released into circulation by tissue damage or haemolysis, and protecting against lipid peroxidation [58–60]. Acute phase proteins can be produced in the mammary gland during weaning when the gland involutes and the re-structuring tissues may then be open to infection [61]. Haptoglobin’s appearance in panda milk at the onset of lactation may, therefore, be protective against infection of the restructuring mammary tissues.

### 4.2 Oligosaccharides

We also observed dramatic changes in oligosaccharides that are considered to be part of the innate immune system. Notable were the trisaccharides sialyllactose (3′-*N*-acetylneuraminyllactose), the concentration of which fell until 20–30 days, and fucosyllactose, which began to increase at that time. These, along with other milk oligosaccharides, appear not to be digested by mammals but instead act to prevent bacterial colonization of the gut by competing with sugar receptors on the surface of pathogens [26,62]. Fucosylated oligosaccharides, for instance, are known to inhibit adhesion of several bacterial pathogens to human cells [63], and sialylated forms have similar activities [64]. Complex oligosaccharides appear to be particularly important in the milks of primates [65], as exemplified by the finding that the infants of breastfeeding human mothers that have low levels of certain oligosaccharides in their milk are susceptible to episodes of diarrhoea [66]. Moreover, fucosylated and other milk oligosaccharides stimulate the growth of certain bifidobacteria which lend competitive protection against pathogens and their toxins [26,62,67–69]. It could, therefore, be that the sequential appearance and disappearance of oligosaccharides in panda milk simultaneously regulate the establishment of a microbiome appropriate for a given stage of altricial development and provide protection against pathogens while the cub’s active immunity develops. It is notable that the changeover period between those oligosaccharides that rose and those that fell corresponded to the time after birth when the protein profile approached maturity.

### 4.3 Other small molecules

Lactose has been found previously in bear milks, albeit usually at low levels [25,70]. Its concentration in giant panda milk fell rapidly after birth (electronic supplementary material, table S4 and figure S3). Lactose is commonly low or absent in milks of the Carnivora (particularly in the suborder Caniformia to which bears belong [1,39,71]), which may mean that lactase synthesis is brief in giant pandas, and that cubs may lose the ability to digest high-lactose milks from about three weeks after birth. Supplementation with artificial milk formulae that are made primarily of cow (or other high-lactose) milk would, therefore, be deleterious to hand-reared cubs.

We found taurine to be persistently elevated in giant panda milk for at least five months, and at higher concentrations than in our comparative bovine and human samples. Similar levels were found in our polar bear milk samples (data not shown). Most mammals can synthesize their own taurine, but taurine-dependency is a feature of some hypercarnivores such as cats, and possibly also polar bears [72], whose requirements are met though carnivory. It is, therefore, conceivable that taurine-dependency occurs more widely in ursids, which merits investigation to ensure adequate nutrition of captive bears and hand-reared cubs. Taurine-dependency in giant pandas would be interesting, given their predominantly vegetarian diet. That said, the panda’s carnivorous habits in the wild have been well documented [73,74].

An unexpected constituent of giant panda milk was phenol sulfate. Its presence corresponded to the 7–14 days anorexic phase during which time the females do not leave the den. One possible explanation for the transience of this compound in the milk is that during starvation gut bacteria begin to scavenge nitrogen from their proteins, the consequence being the release of toxic phenols which are neutralized by conversion into sulfates by gut cells [75], and hence percolate into the milk. It is, therefore, reasonable to expect that, once normal nutritional conditions return, phenol sulfate would disappear from the milk, as we found to be the case.

### 4.4 Evolutionary considerations

One hypothesis with which to explain the production of altricial neonates addresses the need for energy conservation during a period of metabolic downregulation in the mother. The bear species that hibernate give birth during this period, but mobilization of maternal fat reserves needed to supply embryos with a predominantly glucose-based trans-placental energy supply is energetically wasteful and unsustainable during hibernation [14]. Milk, on the other hand, is an efficient means for delivery of fats that does not carry such metabolic costs [2,3]. The confounding factor is that hibernation is thought to have arisen only once in bears, and is found in polar, brown and black bears [34,36]), while giant pandas and sloth, sun and Andean bears do not hibernate. Assuming that none of the non-hibernating species had hibernating ancestors, then there is still no answer as to why bears have such altricial young, and why this characteristic is so particularly pronounced in giant pandas. In other aspects of reproduction, bears are not particularly unusual—within the Carnivora, for example, the duration of dependence by bear cubs on milk is relatively long, but not remarkably so [2], and the type of placenta they have (endotheliochorial) is not restricted to species with altricial neonates [16,76].

In this study, we turned to giant pandas as representatives of extreme altriciality even among Ursidae, to test our hypothesis that, compared with other eutherians, maturation of colostrum to mature milk would be prolonged. We did indeed find a slow transition in the types of proteins and micronutrients present in giant panda milk over time. Comparative detail is, however, available for few other species. Our findings suggest that early lactation in bears may be modified to support the development of unusually immature neonates, albeit not to the extreme of marsupials [8,9]. We did not, for instance, observe in giant panda milk the marked and sequential changeover of protein types that is observed in marsupial milks [4,9,10]. The proteins we identified in panda milks were only those shared among marsupials and eutherians; we did not identify any that were similar to those unique to marsupials. So, the transition in giant pandas is essentially a prolongation of the pattern in other eutherians rather than a convergence to that of marsupials.

Whether giving birth to highly altricial young by bears is a special adaptation or is the retention of a primitive or ancestral reproductive strategy is unresolved, but the consequence may have been the evolution of a slow transition from colostrum to mature milk encompassing a prolonged and changing supply of innate and adaptive immune protective factors. The kinetics of this transition may be unique or at least highly unusual among eutherian mammals, although there is currently insufficient coverage of mammalian lineages to be sure. The analysis presented here is as yet unprecedented in the literature for any other species of eutherian mammal, though it is already clear that in well-studied species the transition is considerably shorter than we find for giant pandas (e.g. camels [18]; pigs [19]; cows [17]). It remains intriguing that even within one group of mammals, the Carnivora, there are species possessing similar placentae and maternal body masses yet giving birth to neonates representing extremes of altriciality or precocity such that, for instance, giant pandas take longer to achieve mature phase lactation than some species of equivalent maternal body masses to bears have an entire lactation cycle (e.g. the Phocidae). Our findings, and similar studies on a broad range of eutherian groups, emphasize and extend our appreciation of the remarkable diversity of eutherian lactation strategies, and illuminate the selective forces involved.

## Supplementary Material

Giant panda milk transition electronic supplementary material.pdf.

## Data accessibility

All data analysed in this study are available online in the electronic supplementary material.

## References

[1] Langer P 2009 Differences in the composition of colostrum and milk in eutherians reflect differences in immunoglobulin transfer. J. Mammal. 90, 332–339. (doi:10.1644/08-mamm-a-071.1)

[2] Langer P 2008 The phases of maternal investment in eutherian mammals. Zoology 111, 148–162. (doi:10.1016/j.zool.2007.06.007) 18222662 10.1016/j.zool.2007.06.007

[3] Skibiel AL, Downing LM, Orr TJ, Hood WR 2013 The evolution of the nutrient composition of mammalian milks. J. Anim. Ecol. 82, 1254–1264. (doi:10.1111/1365-2656.12095) 23895187 10.1111/1365-2656.12095

[4] Brennan AJ, Sharp JA, Lefevre C, Topcic D, Auguste A, Digby M, Nicholas KR 2007 The tammar wallaby and fur seal: models to examine local control of lactation. J. Dairy Sci. 90, E66–E75. (doi:10.3168/jds.2006-483) 17517753 10.3168/jds.2006-483

[5] Schulz TM, Bowen WD 2004 Pinniped lactation strategies: evaluation of data on maternal and offspring life history traits. Mar. Mamm. Sci. 20, 86–114. (doi:10.1111/j.1748-7692.2004.tb01142.x)

[6] Kovacs KM, Lavigne DM 1992 Maternal investment in otariid seals and walruses. Can. J. Zool. 70, 1953–1964. (doi:10.1139/z92-265)

[7] Oftedal OT, Boness DJ, Tedman RA 1987 The behavior, physiology, and anatomy of lactation in the Pinnipedia. Curr. Mammal. 1, 175–245. (doi:10.1007/978-1-4757-9909-5_6)

[8] Adamski FM, Demmer J 2000 Immunological protection of the vulnerable marsupial pouch young: two periods of immune transfer during lactation in *Trichosurus vulpecula* (brushtail possum). Dev. Compar. Immunol. 24, 491–502. (doi:10.1016/s0145-305x(00)00012-4) 10.1016/s0145-305x(00)00012-4 10785274

[9] Simpson KJ, Nicholas KR 2002 The comparative biology of whey proteins. J. Mammary Gland Biol. Neoplasia 7, 313–326. (doi:10.1023/a:1022856801175) 12751894 10.1023/a:1022856801175

[10] Trott JF, Simpson KJ, Moyle RLC, Hearn CM, Shaw G, Nicholas KR, Renfree MB 2003 Maternal regulation of milk composition, milk production, and pouch young development during lactation in the tammar wallaby (*Macropus eugenii*). Biol. Reprod. 68, 929–936. (doi:10.1095/biolreprod.102.005934) 12604644 10.1095/biolreprod.102.005934

[11] Roopenian DC, Akilesh S 2007 FcRn: the neonatal Fc receptor comes of age. Nat. Rev. Immunol. 7, 715–725. (doi:10.1038/nri2155) 17703228 10.1038/nri2155

[12] Simister NE 2003 Placental transport of immunoglobulin G. Vaccine 21, 3365–3369. (doi:10.1016/s0264-410x(03)00334-7) 12850341 10.1016/s0264-410x(03)00334-7

[13] Baumrucker CR, Bruckmaier RM 2014 Colostrogenesis: IgG_1_ transcytosis mechanisms. J. Mammary Gland Biol. Neoplasia 19, 103–117. (doi:10.1007/s10911-013-9313-5) 24474529 10.1007/s10911-013-9313-5

[14] Ramsay MA, Dunbrack RL 1986 Physiological constraints on life-history phenomena—the example of small bear cubs at birth. Am. Nat. 127, 735–743. (doi:10.1086/284522)

[15] Loeffler IK, Montali RJ, Rideout BA 2006 Diseases and pathology of giant pandas. In Giant pandas in captivity: biology and medicine (eds Wildt DE, Zhang A, Zhang H, Janssen DL, Ellis S), pp. 377–409. Cambridge, UK: Cambridge University Press.

[16] Gundling WE, Wildman DE 2015 A review of inter- and intraspecific variation in the eutherian placenta. Phil. Trans. R. Soc. B 370, 20140072 (doi:10.1098/rstb.2014.0072) 25602076 10.1098/rstb.2014.0072 PMC4305173

[17] Thomas FC 2015 Acute phase proteins, proteomics and metabolomics in the diagnosis of bovine mastitis. PhD thesis. University of Glasgow, Glasgow, UK.

[18] Zhang H, Yao J, Zhao D, Liu H, Li J, Guo M 2005 Changes in chemical composition of Alxa bactrian camel milk during lactation. J. Dairy Sci. 88, 3402–3410. (doi:10.3168/jds.S0022-0302(05)73024-1) 16162513 10.3168/jds.S0022-0302(05)73024-1

[19] Ogawa S, Tsukahara T, Nishibayashi R, Nakatani M, Okutani M, Nakanishi N, Ushida K, Inoue R 2014 Shotgun proteomic analysis of porcine colostrum and mature milk. Anim. Sci. J. 85, 440–448. (doi:10.1111/asj.12165) 24450292 10.1111/asj.12165

[20] Liu XZ, Li MX, Yu JQ, Zhang ZH, Huang XM, Lan JC, Yang Z 2005 Composition of captive giant panda milk. Zoo Biol. 24, 393–398. (doi:10.1002/zoo.20056)

[21] Liu XZ, Yu JQ, Li MX, Huang XM, Li GH 2003 Nutrient content of the milk of captive giant pandas. Acta Zool. Sin. 49, 494–500.

[22] Liu X, Li M, Yu J 2008 Nutrient composition and changes in the milk of captive giant pandas. Chin. J. Appl. Environ. Biol. 14, 220–224.

[23] Nakamura T *et al.* 2003 Composition and oligosaccharides of a milk sample of the giant panda, *Ailuropoda melanoleuca*. Compar. Biochem. Physiol. B 135, 439–448. (doi:10.1016/s1096-4959(03)00093-9) 10.1016/s1096-4959(03)00093-9 12831764

[24] Liu X, Yu J, Li X, Li S, Zhong S, Zhang W, Shi S 1996 Nutrient analysis of the milk from captive giant panda and discussion on the nutrient requirement for its baby. Chin. J. Appl. Environ. Biol. 2, 382–386.

[25] Hudson GJ, Bailey PA, John PMV, Monsalve L, Delcampo ALG, Taylor DC, Kay JDS 1984 Composition of milk from *Ailuropoda melanoleuca*, the giant panda. Vet. Rec. 115, 252 (doi:10.1136/vr.115.10.252) 6495573 10.1136/vr.115.10.252

[26] Zivkovic AM, German JB, Lebrilla CB, Mills DA 2011 Human milk glycobiome and its impact on the infant gastrointestinal microbiota. Proc. Natl Acad. Sci. USA 108, 4653–4658. (doi:10.1073/pnas.1000083107) 20679197 10.1073/pnas.1000083107 PMC3063602

[27] Musilova S, Rada V, Vlkova E, Bunesova V 2014 Beneficial effects of human milk oligosaccharides on gut microbiota. Beneficial Microbes 5, 273–283. (doi:10.3920/bm2013.0080) 24913838 10.3920/BM2013.0080

[28] Zhu L, Wu Q, Dai J, Zhang S, Wei F 2011 Evidence of cellulose metabolism by the giant panda gut microbiome. Proc. Natl Acad. Sci. USA 108, 17 714–17 719. (doi:10.1073/pnas.1017956108) 10.1073/pnas.1017956108 PMC3203778 22006317

[29] Fang W, Fang ZM, Zhou P, Chang F, Hong YZ, Zhang XC, Peng H, Xiao YZ 2012 Evidence for lignin oxidation by the giant panda fecal microbiome. PLoS ONE 7, e50312 (doi:10.1371/journal.pone.0050312) 23209704 10.1371/journal.pone.0050312 PMC3508987

[30] Daneshvar H, Wyllie S, Phillips S, Hagan P, Burchmore R 2012 Comparative proteomics profiling of a gentamicin-attenuated *Leishmania infantum* cell line identifies key changes in parasite thiol-redox metabolism. J. Proteomics 75, 1463–1471. (doi:10.1016/j.jprot.2011.11.018) 22154982 10.1016/j.jprot.2011.11.018

[31] Braceland M *et al.* 2013 The serum proteome of Atlantic salmon, *Salmo salar*, during pancreas disease (PD) following infection with salmonid alphavirus subtype 3 (SAV3). J. Proteomics 94, 423–436. (doi:10.1016/j.jprot.2013.10.016) 24145143 10.1016/j.jprot.2013.10.016 PMC3878379

[32] Eizirik E, Murphy WJ, Koepfli KP, Johnson WE, Dragoo JW, Wayne RK, O’Brien SJ 2010 Pattern and timing of diversification of the mammalian order Carnivora inferred from multiple nuclear gene sequences. Mol. Phylogenet. Evol. 56, 49–63. (doi:10.1016/j.ympev.2010.01.033) 20138220 10.1016/j.ympev.2010.01.033 PMC7034395

[33] Vislobokova IA 2013 On the origin of Cetartiodactyla: comparison of data on evolutionary morphology and molecular biology. Paleontol. J. 47, 321–334. (doi:10.1134/s003103011303012x)

[34] Kutschera VE, Bidon T, Hailer F, Rodi JL, Fain SR, Janke A 2014 Bears in a forest of gene trees: phylogenetic inference is complicated by incomplete lineage sorting and gene flow. Mol. Biol. Evol. 31, 2004–2017. (doi:10.1093/molbev/msu186) 24903145 10.1093/molbev/msu186 PMC4104321

[35] Cahill JA, Stirling I, Kistler L, Salamzade R, Ersmark E, Fulton TL, Stiller M, Green RE, Shapiro B 2015 Genomic evidence of geographically widespread effect of gene flow from polar bears into brown bears. Mol. Ecol. 24, 1205–1217. (doi:10.1111/mec.13038) 25490862 10.1111/mec.13038 PMC4409089

[36] Pages M, Calvignac S, Klein C, Paris M, Hughes S, Hanni C 2008 Combined analysis of fourteen nuclear genes refines the Ursidae phylogeny. Mol. Phylogenet. Evol. 47, 73–83. (doi:10.1016/j.ympev.2007.10.019) 18328735 10.1016/j.ympev.2007.10.019

[37] Kaetzel CS 2005 The polymeric immunoglobulin receptor: bridging innate and adaptive immune responses at mucosal surfaces. Immunol. Rev. 206, 83–99. (doi:10.1111/j.0105-2896.2005.00278.x) 16048543 10.1111/j.0105-2896.2005.00278.x

[38] Holt C, Carver JA, Ecroyd H, Thorn DC 2013 Invited review: caseins and the casein micelle: their biological functions, structures, and behavior in foods. J. Dairy Sci. 96, 6127–6146. (doi:10.3168/jds.2013-6831) 23958008 10.3168/jds.2013-6831

[39] Jenness R, Holt C 1987 Casein and lactose concentrations in milk of 31 species are negatively correlated. Experientia 43, 1015–1018. (doi:10.1007/bf01952224) 3653340 10.1007/BF01952224

[40] Dotz V, Rudloff S, Meyer C, Lochnit G, Kunz C 2015 Metabolic fate of neutral human milk oligosaccharides in exclusively breast-fed infants. Mol. Nutr. Food Res. 59, 355–364. (doi:10.1002/mnfr.201400160) 25330044 10.1002/mnfr.201400160

[41] Pacheco AR, Barile D, Underwood MA, Mills DA 2015 The impact of the milk glycobiome on the neonate gut microbiota. Annu. Rev. Anim. Biosci. 3, 419–445. (doi:10.1146/annurev-animal-022114-111112) 25387230 10.1146/annurev-animal-022114-111112 PMC4349412

[42] ten Bruggencate SJM, Bovee-Oudenhoven IMJ, Feitsma AL, van Hoffen E, Schoterman MHC 2014 Functional role and mechanisms of sialyllactose and other sialylated milk oligosaccharides. Nutr. Rev. 72, 377–389. (doi:10.1111/Nure.12106) 24828428 10.1111/nure.12106

[43] Yu Y *et al.* 2014 Human milk contains novel glycans that are potential decoy receptors for neonatal rotaviruses. Mol. Cell. Proteomics 13, 2944–2960. (doi:10.1074/mcp.M114.039875) 25048705 10.1074/mcp.M114.039875 PMC4223483

[44] Brandtzaeg P 2013 Secretory IgA: designed for anti-microbial defense. Front. Immunol. 4, 222 (doi:10.3389/fimmu.2013.00222) 23964273 10.3389/fimmu.2013.00222 PMC3734371

[45] Rogier EW, Frantz AL, Bruno MEC, Wedlund L, Cohen DA, Stromberg AJ, Kaetzel CS 2014 Secretory antibodies in breast milk promote long-term intestinal homeostasis by regulating the gut microbiota and host gene expression. Proc. Natl Acad. Sci. USA 111, 3074–3079. (doi:10.1073/pnas.1315792111) 24569806 10.1073/pnas.1315792111 PMC3939878

[46] Butler JE, Kehrli ME 2005 Immunoglobulins and immunocytes in the mammary gland and its secretions. In Mucosal immunology, 3rd edn (eds J Mestecky, ME Lamm, W Strober, J Bienenstock, JR McGhee, L Mayer), pp. 1763–1793. San Diego, CA: Elsevier Academic Press Inc.

[47] Farrell HM *et al.* 2004 Nomenclature of the proteins of cows’ milk—sixth revision. J. Dairy Sci. 87, 1641–1674. (doi:10.3168/jds.S0022-0302(04)73319-6) 15453478 10.3168/jds.S0022-0302(04)73319-6

[48] Le Maux S, Bouhallab S, Giblin L, Brodkorb A, Croguennec T 2014 Bovine β-lactoglobulin/fatty acid complexes: binding, structural, and biological properties. Dairy Sci. Technol. 94, 409–426. (doi:10.1007/s13594-014-0160-y) 25110551 10.1007/s13594-014-0160-y PMC4121524

[49] Kontopidis G, Holt C, Sawyer L 2004 Invited review: β-lactoglobulin: binding properties, structure, and function. J. Dairy Sci. 87, 785–796. (doi:10.3168/jds.S0022-0302(04)73222-1) 15259212 10.3168/jds.S0022-0302(04)73222-1

[50] Holt C, Carver JA 2012 Darwinian transformation of a ‘scarcely nutritious fluid’ into milk. J. Evol. Biol. 25, 1253–1263. (doi:10.1111/j.1420-9101.2012.02509.x) 22524460 10.1111/j.1420-9101.2012.02509.x

[51] Lenton S, Nylander T, Teixeira SCM, Holt C 2015 A review of the biology of calcium phosphate sequestration with special reference to milk. Dairy Sci. Technol. 95, 3–14. (doi:10.1007/s13594-014-0177-2) 25632319 10.1007/s13594-014-0177-2 PMC4302223

[52] Holt C 2013 Unfolded phosphopolypeptides enable soft and hard tissues to coexist in the same organism with relative ease. Curr. Opin. Struct. Biol. 23, 420–425. (doi:10.1016/j.sbi.2013.02.010) 23622834 10.1016/j.sbi.2013.02.010

[53] Loeffler IK 2007 Veterinary considerations for rehabilitation and release of bear cubs. In Proc. of the Int. Workshop on the Rehabilitation, Release and Monitoring of Orphaned Bear Cubs. Bubonitsy, Russia: International Fund for Animal Welfare.

[54] Lintzenich BA, Ward AM, Edwards MS, Griffin ME, Robbins CT 2006 Polar bear nutrition guidelines. American Zoological Association Bear Taxon Advisory Group.

[55] Landberg E, Huang YP, Stromqvist M, Mechref Y, Hansson L, Lundblad A, Novotny MV, Pahlsson P 2000 Changes in glycosylation of human bile-salt-stimulated lipase during lactation. Arch. Biochem. Biophys. 377, 246–254. (doi:10.1006/abbi.2000.1778) 10845701 10.1006/abbi.2000.1778

[56] Andersson E-L, Hernell O, Bläckberg L, Fält H, Lindquist S 2011 BSSL and PLRP2: key enzymes for lipid digestion in the newborn examined using the Caco-2 cell line. J. Lipid Res. 52, 1949–1956. (doi:10.1194/jlr.M015685) 21865348 10.1194/jlr.M015685 PMC3196226

[57] Lombardo D 2001 Bile salt-dependent lipase: its pathophysiological implications. Biochim. Biophys. Acta 1533, 1–28. (doi:10.1016/s1388-1981(01)00130-5) 11514232 10.1016/s1388-1981(01)00130-5

[58] Andersen CBF, Torvund-Jensen M, Nielsen MJ, Pinto de Oliveira CL, Hersleth H-P, Andersen NH, Pedersen JS, Andersen GR, Moestrup SK 2012 Structure of the haptoglobin–haemoglobin complex. Nature 489, 456–459. (doi:10.1038/nature11369) 22922649 10.1038/nature11369

[59] Ceron JJ, Eckersall PD, Martinez-Subiela S 2005 Acute phase proteins in dogs and cats: current knowledge and future perspectives. Vet. Clin. Pathol. 34, 85–99. (doi:10.1111/j.1939-165X.2005.tb00019.x) 15902658 10.1111/j.1939-165x.2005.tb00019.x

[60] Eaton JW, Brandt P, Mahoney JR, Lee JT 1982 Haptoglobin—a natural bacteriostat. Science 215, 691–693. (doi:10.1126/science.7036344) 7036344 10.1126/science.7036344

[61] Stein T *et al.* 2004 Involution of the mouse mammary gland is associated with an immune cascade and an acute-phase response, involving LBP, CD14 and STAT3. Breast Cancer Res. 6, R75–R91. (doi:10.1186/bcr753) 14979920 10.1186/bcr753 PMC400652

[62] Hinde K, Lewis ZT 2015 Mother’s littlest helpers. Science 348, 1427–1428. (doi:10.1126/science.aac7436) 26113704 10.1126/science.aac7436

[63] Ruiz-Palacios GM, Cervantes LE, Ramos P, Chavez-Munguia B, Newburg DS 2003 *Campylobacter jejuni* binds intestinal H(O) antigen (Fuc α1, 2Gal β1, 4GlcNAc), and fucosyloligosaccharides of human milk inhibit its binding and infection. J. Biol. Chem. 278, 14 112–14 120. (doi:10.1074/jbc.M207744200) 12562767 10.1074/jbc.M207744200

[64] Murakami Y, Higuchi N, Nakamura H, Yoshimura F, Oppenheim FG 2002 *Bacteroides forsythus* hemagglutinin is inhibited by *N*-acetylneuraminyllactose. Oral Microbiol. Immunol. 17, 125–128. (doi:10.1046/j.0902-0055.2001.00093.x) 11929561 10.1046/j.0902-0055.2001.00093.x

[65] Tao NA, Wu SA, Kim J, An HJ, Hinde K, Power ML, Gagneux P, German JB, Lebrilla CB 2011 Evolutionary glycomics: characterization of milk oligosaccharides in primates. J. Proteome Res. 10, 1548–1557. (doi:10.1021/pr1009367) 21214271 10.1021/pr1009367 PMC3070053

[66] Morrow AL *et al.* 2004 Human milk oligosaccharides are associated with protection against diarrhea in breast-fed infants. J. Pediatr. 145, 297–303. (doi:10.1016/j.jpeds.2004.04.054) 15343178 10.1016/j.jpeds.2004.04.054

[67] Yu ZT, Chen C, Newburg DS 2013 Utilization of major fucosylated and sialylated human milk oligosaccharides by isolated human gut microbes. Glycobiology 23, 1281–1292. (doi:10.1093/glycob/cwt065) 24013960 10.1093/glycob/cwt065 PMC3796377

[68] Hoeflinger JL, Davis SR, Chow J, Miller MJ 2015 *In vitro* impact of human milk oligosaccharides on Enterobacteriaceae growth. J. Agric. Food Chem. 63, 3295–3302. (doi:10.1021/jf505721p) 25748944 10.1021/jf505721p

[69] Sela DA *et al.* 2008 The genome sequence of *Bifidobacterium longum* subsp. *infantis* reveals adaptations for milk utilization within the infant microbiome. Proc. Natl Acad. Sci. USA 105, 18 964–18 969. (doi:10.1073/pnas.0809584105) 10.1073/pnas.0809584105 PMC2596198 19033196

[70] Nakamura T, Kawase H, Kimura K, Watanabe Y, Ohtani M, Arai I, Urashima T 2003 Concentrations of sialyloligosaccharides in bovine colostrum and milk during the prepartum and early lactation. J. Dairy Sci. 86, 1315–1320. (doi:10.3168/jds.S0022-0302(03)73715-1) 12741556 10.3168/jds.S0022-0302(03)73715-1

[71] Uemura Y, Takahashi S, Senda A, Fukuda K, Saito T, Oftedal OT, Urashima T 2009 Chemical characterization of milk oligosaccharides of a spotted hyena (*Crocuta crocuta*). Comp. Biochem. Physiol. A 152, 158–161. (doi:10.1016/j.cbpa.2008.09.013) 10.1016/j.cbpa.2008.09.013 18840538

[72] Hedberg GE, Derocher AE, Andersen M, Rogers QR, DePeters EJ, Loennerdal B, Mazzaro L, Chesney RW, Hollis B 2011 Milk composition in free-ranging polar bears (*Ursus maritimus*) as a model for captive rearing milk formula. Zoo Biol. 30, 550–565. (doi:10.1002/zoo.20375) 21246608 10.1002/zoo.20375

[73] Schaller GB, Hu J, Pan W, Zhu J 1985 The giant pandas of Wolong. Chicago, IL: University of Chicago Press.

[74] Lindburg D, Baragona K (eds) 2004 Giant pandas: biology and conservation. Berkeley, CA: University of California Press.

[75] Kikuchi K, Itoh Y, Tateoka R, Ezawa A, Murakami K, Niwa T 2010 Metabolomic search for uremic toxins as indicators of the effect of an oral sorbent AST-120 by liquid chromatography/tandem mass spectrometry. J. Chromatogr. B 878, 2997–3002. (doi:10.1016/j.jchromb.2010.09.006) 10.1016/j.jchromb.2010.09.006 20870466

[76] Mess A, Carter AM 2007 Evolution of the placenta during the early radiation of placental mammals. Comp. Biochem. Physiol. A 148, 769–779. (doi:10.1016/j.cbpa.2007.01.029) 10.1016/j.cbpa.2007.01.029 17347003

