# Supplementary material for: Prolonged transition time between colostrum and mature milk in a bear, the giant panda, Ailuropoda melanoleuca
Source: R Soc Open Sci. 2015 Oct 21;2(10):150395. doi: 10.1098/rsos.150395 (PMC4632522; doi:10.1098/rsos.150395)
Supplement: Giant panda milk transition electronic supplementary material.pdf. [file rsos150395supp1.pdf]

Prolonged transition time between colostrum and mature milk in a bear, the giant panda, *Ailuropoda melanoleuca*

Kate Griffiths[1], Rong Hou[2], Hairui Wang[2], Zhihe Zhang[2], Liang Zhang[2], Tong Zhang[3], David G. Watson[3], Richard J.S. Burchmore[4], I. Kati Loeffler[2*] and Malcolm W. Kennedy[1] .

[1] Institute of Biodiversity, Animal Health and Comparative Medicine, Graham Kerr Building, College of Medical, Veterinary and Life Sciences, University of Glasgow, Glasgow G12 8QQ, Scotland, UK.
[2] The Sichuan Key Laboratory for Conservation Biology on Endangered Wildlife; Chengdu Research Base of Giant Panda Breeding, 1375 Panda Road, Northern Suburb, Chengdu, Sichuan Province, P.R. China, 610081.
[3] Strathclyde Institute of Pharmacy and Biomedical Sciences, University of Strathclyde, 161 Cathedral Street, Glasgow G4 0RE, Scotland, UK.
[4] Institute of Infection, Immunity and Inflammation and Glasgow Polyomics, University of Glasgow, Garscube Campus, Glasgow G12 1QH, Scotland, UK.
* Present address: International Fund for Animal Welfare, 290 Summer Street, Yarmouth Port, MA 02675, USA.

Electronic supplementary material

Figure S1. Altriciality of the neonates of giant pandas.

Table S1. Reproductive history and milk sampling times of giant pandas from whom samples were collected.

Figure S2. Changes in protein profiles of giant panda milk with time after parturition – preparative two-dimensional gels.

Table S2. Identification of the proteins isolated from bands excised from the two-dimensional gels shown in Figure S2.

Table S3. Proteins in giant panda milk. Identification of the proteins isolated from bands excised from the one-dimensional gels shown in Figure 3 in the main paper.

Table S4. Relative abundance of the 10 most common small molecules in giant panda milks in colostrum and mature milk, compared with mature phase human and cow milks.

Table S5. Milk oligosaccharides reported in this and previous studies on giant pandas and other species of bear, and their post-partum trends in giant panda milk.

Figure S3. Diversity in the concentration time courses of selected giant panda milk oligosaccharides with time after birth.

References for electronic supplementary material

Figure S1. Altriciality of the neonates of giant pandas. Photographs of newly born giant panda cubs with their mothers illustrating both their highly altricial stage of development, the relative size of mothers and cubs, and thus the considerable degree of maternal care required. Images courtesy of Chengdu Research Centre for Giant Panda Breeding.

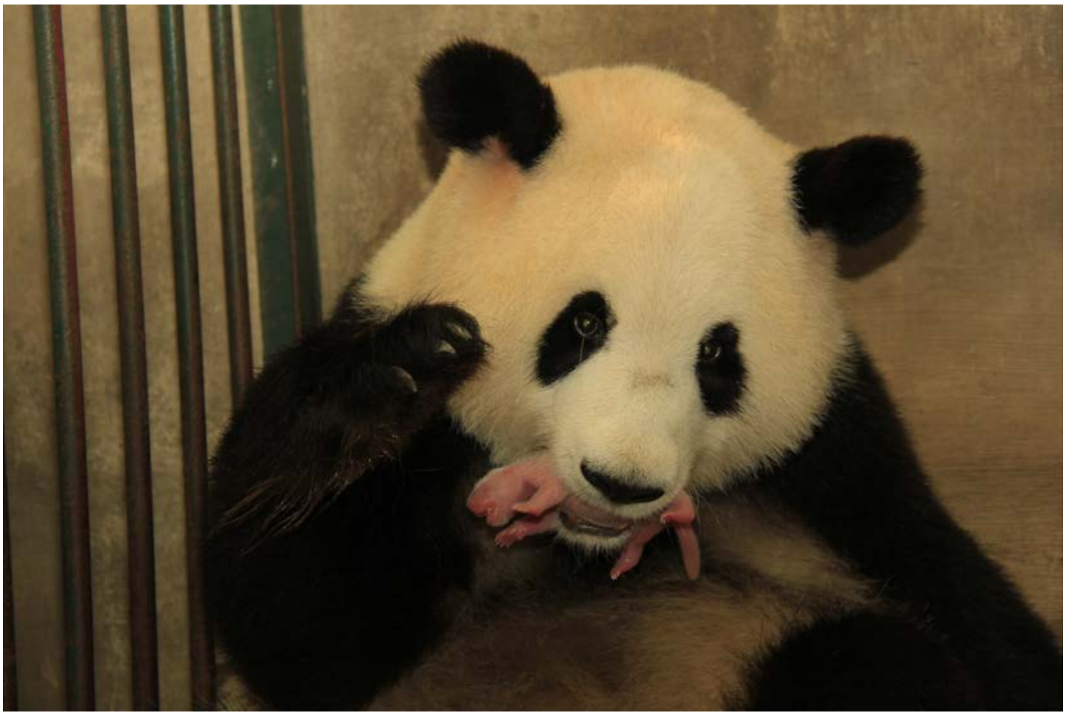

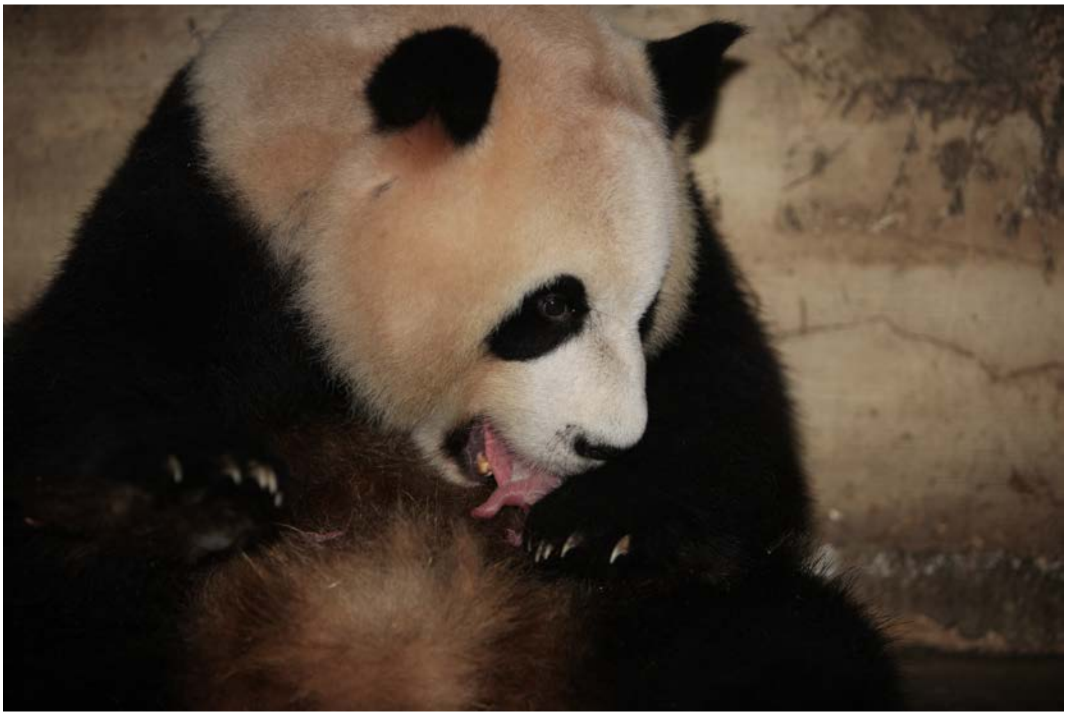

Table S1. Reproductive history and milk sampling times of giant pandas from whom samples were collected. All mothers and cubs were born in captivity. ISN, International Studbook Number. Parturition dates in bold indicate the beginning of the lactation period from which samples were collected.

Name, ISN & date of birth	Parturition history	Milk sample collection dates	Days post-partum
Ya Ya<br>362<br>24 Aug 1990	9 Sept 1997, twins; 1 died same day<br>9 Sept 1999, twins<br>20 Aug 2001, twins<br>28 Aug 2002, twins<br>6 Sept 2003, single<br>30 Aug 2005, single<br>**19 Aug 2006, single**<br>24 Aug 2008, single<br>10 Sept 2010, single	13 Jan 2007<br>18 Jan 2007<br>23 Jan 2007<br>29 Jan 2007<br>31 Jan 2007<br>07 Feb 2007	147<br>152<br>157<br>163<br>165<br>172
Li Li<br>387<br>03 Sept 1992	12 July 2001, twins, died 19 Sept & 17 Oct, 2001<br>08 July 2002, single, died 08 July 2002<br>**03 Aug 2007, single, died same day**<br>19 July 2009, twins<br>**24 July 2011, single**<br>**28 July 2012, twins, 1 died same day**	07 Aug 2007	4
		25 July 2011<br>26 July 2011<br>28 July 2011<br>29 July 2011<br>30 July 2011<br>31 July 2011<br>01 Aug 2011<br>02 Aug 2011<br>03 Aug 2011<br>05 Aug 2011<br>06 Aug 2011<br>07 Aug 2011<br>08 Aug 2011<br>09 Aug 2011<br>10 Aug 2011<br>11 Aug 2011<br>12 Aug 2011<br>14 Aug 2011<br>15 Aug 2011	1<br>2<br>4<br>5<br>6<br>7<br>8<br>9<br>10<br>12<br>13<br>14<br>15<br>16<br>17<br>18<br>19<br>21<br>22
		29 July 2012<br>30 July 2012<br>31 July 2012<br>1 Aug 2012<br>2 Aug 2012<br>3 Aug 2012<br>4 Aug 2012<br>5 Aug 2012<br>6 Aug 2012<br>8 Aug 2012<br>9 Aug 2012	1<br>2<br>3<br>4<br>5<br>6<br>7<br>8<br>9<br>11<br>12

Name, ISN & date of birth	Parturition history	Milk sample collection dates	Days post-partum
		10 Aug 2012<br>11 Aug 2012<br>12 Aug 2012<br>13 Aug 2012<br>15 Aug 2012<br>16 Aug 2012	13<br>14<br>15<br>16<br>18<br>19
Shu Qing<br>480<br>03 Aug 1999	26 Aug 2004, single<br>**05 July 2007, twins**<br>04/05 Aug 2008, twins	08 July 2007<br>20 Aug 2007<br><br>10 Dec 2007	3<br>46<br><br>158
Qi Zhen<br>490<br>04 Sept 1999	**07 Aug 2006, twins**<br>10 Aug 2008, twins<br>06 Aug 2010, twins<br>04 Aug 2011, twins	12 Aug 2006<br>14 Aug 2006<br>13 Aug 2006<br>15 Aug 2006<br>10 Jan 2007<br>11 Jan 2007<br>14 Jan 2007<br>16 Jan 2007	5<br>7<br>6<br>8<br>156<br>157<br>160<br>162
Yuan Yuan<br>561<br>01 Aug 2003	06 Sept 2008, single<br>15 Aug 2010, twins<br>**25 Aug 2012, twins, 1 died same day**	26 Aug 2012<br>27 Aug 2012<br>28 Aug 2012<br>29 Aug 2012<br>01 Sept 2012<br>04 Sept 2012<br>05 Sept 2012<br>08 Sept 2012<br>09 Sept 2012<br>10 Sept 2012<br>13 Sept 2012<br>16 Sept 2012<br>17 Sept 2012<br>19 Sept 2012<br>20 Sept 2012<br>21 Sept 2012<br>23 Sept 2012<br>24 Sept 2012<br>25 Sept 2012<br>01 Oct 2012<br>10 Oct 2012	1<br>2<br>3<br>4<br>7<br>10<br>11<br>14<br>15<br>16<br>19<br>22<br>23<br>25<br>26<br>27<br>29<br>30<br>31<br>37<br>46
Xiao Yatou<br>635<br>13 Aug 2006	**12 Aug 2012, single**	16 Aug 2012<br>24 Aug 2012<br>25 Aug 2012<br>26 Aug 2012	4<br>12<br>13<br>14

Name, ISN & date of birth	Parturition history	Milk sample collection dates	Days post-partum
		27 Aug 2012	15
		30 Aug 2012	17
		03 Sept 2012	22
		13 Sept 2012	32
		14 Sept 2012	33
		17 Sept 2012	36
		18 Sept 2012	37
		19 Sept 2012	38
		20 Sept 2012	39
		21 Sept 2012	40
		30 Sept 2012	49
		01 Oct 2012	50
		09 Oct 2012	58

Figure S2. Changes in protein profiles of giant panda milk with time after parturition – preparative two-dimensional gels. Preparative two-dimensional gel electrophoresis of milk sampled on day1 (A; panda Li Li) and on day 152 (B; panda Ya Ya). The numbered protein spots were excised and subjected to tryptic digestion and mass spectrometry, after which peptide mass fingerprints were used to interrogate the giant panda genome with MASCOT software. Identifications of the numbered protein spots and comments are given in table S2. These identifications were combined with identifications from bands excised from 1-D gels to provide the protein identifications in Figure 3 of the main paper and the associated tables 1 and S3. First dimension (IEF) on IPG strips pH 3–10 and the second dimension on 4–12% gradient SDS-PAGE gels. The protein spots were visualized by Coomassie blue staining.

A

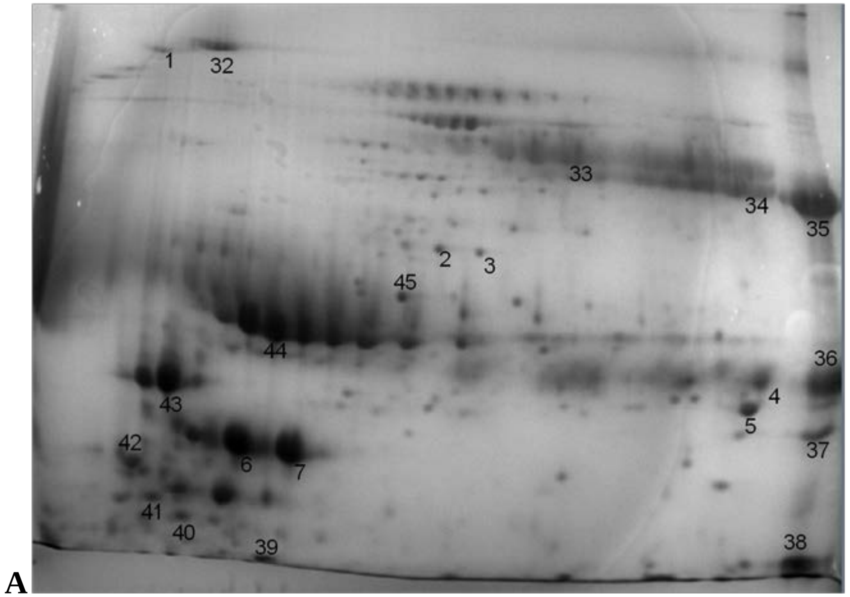

B

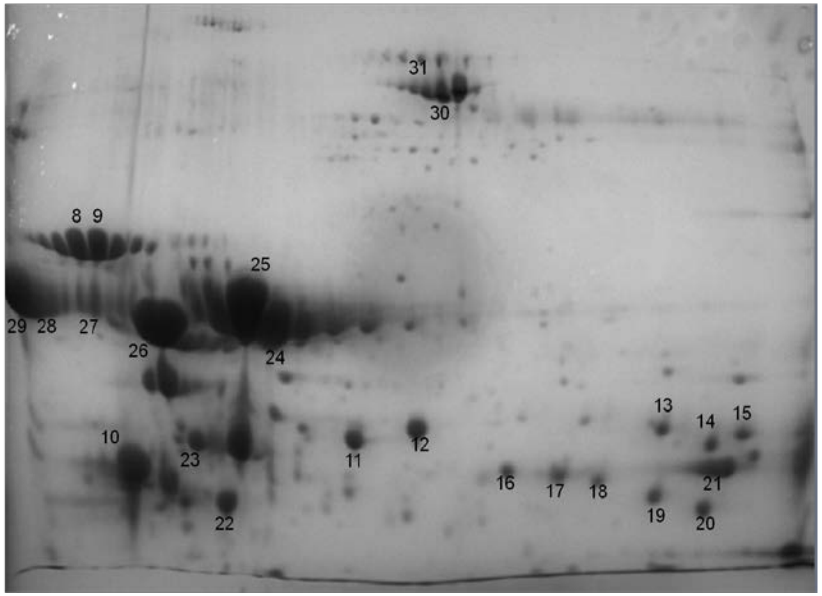

Table S2. Identification of the proteins isolated from bands excised from the two-dimensional gels shown in Figure S2. Milks sampled from panda Li Li 1 day and from Ya Ya 152 days after parturition. The putative functions and comments are drawn from a variety of sources including NCBI and UniProtKB/Swiss-Prot databases.

**Spot number (day of sample)** [a]	**Protein** [b]	**Accession number** [c]	**Number of matches (unique peptides)** [d]	**Mascot score** [e]	**Putative function and comments** [f]
1 (1)	Bile salt-activated lipase	gi|301777998	67(36)	1286	Presumed to assist with digestion of lipids, triglycerides in particular.
2 (1)	Polymeric immunoglobulin receptor	gi|301767800	230(122)	4557	Receptor for IgA and IgM mediating secretion, part of which (secretory piece) binds to IgA to protect it against proteolytic cleavage in intestine.
3 (1)	Polymeric immunoglobulin receptor	gi|301767800	230(122)	4160	Receptor for IgA and IgM mediating secretion, part of which (secretory piece) binds to IgA to protect it against proteolytic cleavage in intestine.
4 (1)	Immunoglobulin λ*	gi|281339129	176(70)	3098	Light chain isoform associated with all immunoglobulin classes.
5 (1)	β-casein	gi|301768260	86(36)	905	Phosphoprotein. Source of amino acids, delivers calcium, phosphate, lipids. Determines the surface properties of the casein micelles.
6 (1)	β-lactoglobulin-1	gi|301778529	68(30)	584	Binds and probably transports retinol (vitamin A), vitamin D, fatty acids including PUFAs [g].
7 (1)	β-lactoglobulin-1	gi|301778533	150(64)	2843	Binds and probably transports retinol (vitamin A), vitamin D, fatty acids including PUFAs [g].
8 (152)	β-lactoglobulin-1	gi|301778529	71(24)	462	Binds and probably transports retinol (vitamin A), vitamin D, fatty acids including PUFAs [g].
9 (152)	Keratin	gi|301779149	26(16)	639	Structural constituent of epidermis.
10 (152)	Whey acidic protein	gi|301777338	93(41)	1247	Function unclear. Innate immunity? Regulates proliferation of mammary epithelial cells?
11 (152)	β-lactoglobulin-2	gi|301778531	276(140)	4222	Binds and probably transports retinol (vitamin A), vitamin D, fatty acids including PUFAs [g].

12 (152)	β-lactoglobulin-2<br>β-lactoglobulin-1	gi|301778531<br>gi|301778533	275(157)<br>71(32)	4850<br>923	Bind and probably transport retinol (vitamin A), vitamin D, fatty acids including PUFAs [g].
13 (152)	β-lactoglobulin-2	gi|301778531	125(2)	131	Binds and probably transports retinol (vitamin A), vitamin D, fatty acids including PUFAs [g].
14 (152)	Keratin	gi|301779149	19(11)	414	Structural constituent of epidermis.
15 (152)	Keratin	gi|301779149	22(14)	467	
16 (152)	Anti-leukoproteinase	gi|301764004	83(27)	663	Acid-stable proteinase inhibitor with strong affinities for trypsin, chymotrypsin, elastase, and cathepsin G.
17 (152)	Anti-leukoproteinase	gi|301764004	64(16)	320	Acid-stable proteinase inhibitor with strong affinities for trypsin, chymotrypsin, elastase, and cathepsin G.
18 (152)	Keratin	gi|301779149	19(10)	458	Structural constituent of epidermis.
19 (152)	Keratin	gi|301779149	12(8)	357	
20 (152)	Keratin	gi|301779149	11(7)	173	
21 (152)	Anti-leukoproteinase	gi|301764004	31(11)	289	Acid-stable proteinase inhibitor with strong affinities for trypsin, chymotrypsin, elastase, and cathepsin G.
22 (152)	α-lactalbumin- 2	gi|301783681	87(40)	1037	Regulatory subunit of lactose synthase. Changes the substrate specificity of galactosyltransferase making glucose a good acceptor substrate for this enzyme enabling LS to synthesize lactose.
23 (152)	β-lactoglobulin-1,<br><br>α-lactalbumin-2	gi|301778533<br><br>gi|301783681	60(23)<br><br>15(6)	575<br><br>137	Binds and probably transports retinol (vitamin A), vitamin D, fatty acids including PUFAs [g].<br><br>Regulatory subunit of lactose synthase. Changes the substrate specificity of galactosyltransferase making glucose a good acceptor substrate for this enzyme enabling LS to synthesize lactose.
24 (152)	β-casein	gi|301768260	73(18)	516	Phosphoprotein. Source of amino acids, delivers calcium, phosphate, lipids. Determines the surface properties of the casein micelles.
25 (152)	β-casein	gi|301768260	67(19)	508	
26 (152)	β-casein	gi|301768260	23(7)	230	
27 (152)	β-casein	gi|301768260	4(2)	92	
28 (152)	Keratin	gi|301775745	2(2)	108	Structural constituent of epidermis.

29 (152)	κ-casein	gi|281347661	3(2)	79	Stabilizes micelle formation, preventing casein precipitation in milk.
30 (152)	Serum albumin	gi|301786252	243(127)	3508	Main protein of plasma. Binds Ca^{2+}, Na^{+}, K^{+}, fatty acids, hormones, bilirubin and drugs. Major zinc transporter of plasma.
31 (152)	Polymeric immunoglobulin receptor	gi|301767800	59(25)	876	Receptor for IgA and IgM mediating secretion, part of which (secretory piece) binds to IgA to protect it against proteolytic cleavage in intestine.
32 (1)	Bile salt-activated lipase	gi|301777998	66(34)	1033	Also termed stimulated- or dependent-lipase. Presumed to assist with digestion of lipids, triglycerides in particular.
33 (1)	Immunoglobulin α	gi|62183972	33(15)	346	Heavy chain of IgA. Secretory antibody. Abundant in secretions (e.g. tears, saliva, bile, milks).
34 (1)	Immunoglobulin γ<br><br>Lactadherin	gi|62183980<br><br>gi|301768371	33(16)<br><br>34(11)	520<br><br>445	Heavy chain of immunoglobulin G (IgG). Antibody. Abundant in serum, less so in secretions.<br><br>Maintains intestinal epithelial homeostasis and the promotion of mucosal healing.
35 (1)	Immunoglobulin γ heavy chain<br><br>Lactadherin	gi|62183978<br><br>gi|301768371	74(38)<br><br>20(13)	1038<br><br>413	Immunoglobulin G (IgG). Antibody. Abundant in serum, less so in secretions.<br><br>Maintains intestinal epithelial homeostasis and the promotion of mucosal healing.
36 (1)	Immunoglobulin λ*	gi|281339129	37(18)	676	Light chain isoform associated with all immunoglobulin subclasses.
37 (1)	No confident prediction				
38 (1)	Lysozyme C	gi|301783675	34(15)	527	Milk isoform of lysozyme. Anti-bacterial.
39 (1)	α-lactalbumin-2	gi|301783681	43(23)	662	Regulatory subunit of lactose synthase. Changes the substrate specificity of galactosyltransferase, making glucose a good acceptor substrate for this enzyme and enabling LS to synthesize lactose.

40 (1)	α-lactalbumin-1,<br><br>β-casein,<br><br>β-lactoglobulin-1	gi|301783679<br><br>gi|301768260<br><br>gi|301778529	14(11)<br><br>35(7)<br><br>7(4)	286<br><br>165<br><br>116	Regulatory subunit of lactose synthase. Changes the substrate specificity of galactosyltransferase making glucose a good acceptor substrate for this enzyme enabling LS to synthesize lactose.<br><br>Phosphoprotein. Source of amino acids, delivers calcium, phosphate, lipids. Determines the surface properties of the casein micelles.<br><br>Binds and probably transports retinol (vitamin A), vitamin D, fatty acids including PUFAs [g].
41 (1)	α-lactalbumin-1	gi|301783679	8(4)	115	Regulatory subunit of lactose synthase. Changes the substrate specificity of galactosyltransferase making glucose a good acceptor substrate for this enzyme, and enabling LS to synthesize lactose.
42 (1)	Whey acidic protein	gi|301777338	23(14)	422	Function unclear. Possibly associated with innate immunity; possible role in regulation of mammary epithelial cell proliferation.
43 (1)	β-lactoglobulin-1	gi|301778533	110(33)	858	Binds and probably transports retinol (vitamin A), vitamin D, fatty acids including PUFAs [g].
44 (1)	β-casein	gi|301768260	85(32)	815	Phosphoprotein. Source of amino acids, delivers calcium, phosphate, lipids. Determines the surface properties of the casein micelles.
45 (1)	Haptoglobin	gi|301776456	105(43)	926	Indicator of infection or inflammation. Acute phase protein. Captures free haemoglobin. Anti-microbial. Also found to high confidence by 2-D gel analysis in 12 hours post-partum sample from the same animal.

[a] Gel bands as indicated in Figure S1.

[b] identification

[c] NCBI Genbank GenInfo accession codes.

[d] Number of peptides found to match with number of peptides unique to this identification in parentheses.

[e] MASCOT (MOWSE) search score where scores greater than 38 are taken to be significant.

[f] Putative functions as listed in NCBI GenBank and UniProtKB databases.

[g] Highly abundant in many mammalian milks. Some species produce only one isoform, and others, such as humans, produce none. PUFAs, polyunsaturated fatty acids.

These identifications were confirmed from 2-D gels carried out on Li Li's milk sampled 12 hours after birth and on day 22.

gi|xxx Accession number from NCBI Genbank database for *Canis lupus familiaris genome* database.

*This identification from BLAST searching of GenBank with nearest similarity in *Canis lupus familiaris* database, but also *Ursus maritimus*; the *A. melanoleuca* NCBI GenBank GeneInfo accession code given here.

Table S3. Proteins in giant panda milk. Identification of the proteins isolated from bands excised from the 1-D SDS-PAGE gels shown in Figure 3 of the main paper, and as an expansion of table 1 of the main paper. The putative functions and comments are drawn from a variety of sources including NCBI and UniProtKB/Swiss-Prot databases.

**Band label** [a]	**Protein identification** [b]	**Accession number** [c]	**Number of matches (unique peptides)** [d]	**Mascot score** [e]	**Putative function and comments** [f]
A	Immunoglobulin λ light chain	gi|281339129*	1(1)	78	Light chain isoform associated with all immunoglobulin subclasses.
	Immunoglobulin μ heavy chain	gi|62183982	2(0)	63	Heavy chain of immunoglobulin M (IgM). Antibody. Abundant in serum, less so in secretions. Abundant in colostrum.**
B	Immunoglobulin α	gi|62183972	20(5)	167	Heavy chain of immunoglobulin A (IgA). Antibody. Abundant in secretions (e.g. tears, saliva, bile, milks).
C	Immunoglobulin α	gi|62183972	45(14)	379	Heavy chain constant region of immunoglobulin A (IgA). Antibody. Abundant in secretions (e.g. tears, saliva, bile, milks).
	Polymeric immunoglobulin receptor	gi|301767800	23(9)	290	Receptor for IgA and IgM mediating secretion, part of which (secretory piece) binds to IgA to protect it against proteolytic cleavage in intestine.
D	Immunoglobulin γ heavy chain	gi|62183980	39(12)	430	Heavy chain of immunoglobulin G (IgG). Antibody. Abundant in serum, less so in secretions.
E	Immunoglobulin γ heavy chain	gi|62183980	15(6)	201	Heavy chain of immunoglobulin G (IgG). Antibody. Abundant in serum, less so in secretions.
F	Bile salt-activated lipase ***	gi|301777998	22(11)	238	Presumed to assist with digestion of lipids, triglycerides in particular. Also termed bile salt stimulated- or dependent-lipase.
G	β-lactoglobulin-2<br>β-lactoglobulin-1	gi|301778529<br>gi|281347108	2(1)<br>7(2)	70<br>69	Bind and probably transport retinol (vitamin A), vitamin D, fatty acids including PUFAs [g].
	κ-casein	gi|281347661	3(1)	46	Stabilizes micelle formation, preventing casein precipitation in milk.
H	β-casein	gi|301768260	39(13)	362	Source of amino acids, delivers calcium, phosphate, lipids.

Band label [a]	Protein identification [b]	Accession number [c]	Number of matches (unique peptides) [d]	Mascot score [e]	Putative function and comments [f]
I	Lactotransferrin	gi|301754041	1(1)	48	Iron binding transport proteins with anti-bacterial properties. Low quality identification.
J	β-lactoglobulin-1	gi|301778533	9(2)	81	Binds and probably transports retinol (vitamin A), vitamin D, fatty acids including PUFAs [g].
K	β-lactoglobulin isoform 1<br><br>Anti-leukoproteinase	gi|301778529<br><br>gi|301778531	9(3)<br><br>6(3)	75<br><br>74	Binds and probably transports retinol (vitamin A), vitamin D, fatty acids including PUFAs [g].<br><br>Acid-stable proteinase inhibitor with strong affinities for trypsin, chymotrypsin, elastase, and cathepsin G.
L	Lysozyme C	gi|301783675	14(6)	265	Milk isoform of lysozyme. Anti-bacterial.
M	Whey acidic protein<br><br>Lysozyme C	gi|301777338<br><br>gi|301783675	4(2)<br><br>1(1)	81<br><br>55	Function unclear. Possibly associated with innate immunity. Possibly plays role in regulation of the proliferation of mammary epithelial cells.<br><br>Milk isoform of lysozyme. Anti-bacterial.
N	Xanthine dehydrogenase	gi|301783299	42(19)	528	Milk fat globule secretion. Innate immunity.
O	Bile salt-activated lipase ***	gi|301777998	22(4)	158	Presumed to assist with digestion of lipids, triglycerides in particular. Also termed bile salt stimulated- or dependent-lipase.
P	Polymeric immunoglobulin receptor	gi|301767800	16(7)	217	Receptor for IgA and IgM mediating secretion, part of which (secretory piece) binds to IgA to protect it against proteolytic cleavage in intestine.
Q	Immunoglobulin γ heavy chain	gi|62183980	20(7)	222	Immunoglobulin G (IgG). Antibody. Abundant in serum, less so in secretions.
R	β-casein	gi|301768260	5(0)	53	Phosphoprotein. Source of amino acids, delivers calcium, phosphate, lipids. Determines the surface properties of the casein micelles.

Band label [a]	Protein identification [b]	Accession number [c]	Number of matches (unique peptides) [d]	Mascot score [e]	Putative function and comments [f]
S	Apolipoprotein D isoform 2	gi|301772170	14(6)	163	Lipid transporter.
	β-casein	gi|301768260	8(2)	62	Source of amino acids, delivers calcium, phosphate, lipids.
	Immunoglobulin λ light chain	gi|73995681*	5(1)	60	Light chain isoform associated with all immunoglobulin subclasses.
T	Immunoglobulin λ light chain	gi|281339129*	41(14)	467	Immunoglobulin G (IgG). Antibody. Abundant in serum, less so in secretions. One of two light-chain isoforms associated with all immunoglobulin subclasses.
U	β-lactoglobulin-1	gi|301778533	7(3)	64	Binds and probably transports retinol (vitamin A), vitamin D, fatty acids including PUFAs [g].

[a] Gel bands as indicated in Figure 3.

[b] Protein identification

[c] NCBI Genbank accession codes and MASCOT data (number of unique peptides, sequence coverage, MASCOT score).

[d] Number of peptides found to match with number of peptides unique to this identification in parenthesis.

[e] MASCOT (MOWSE) search score where scores greater than 38 are taken to be significant.

[f] Putative functions as listed in NCBI GenBank and UniProtKB databases.

[g] PUFAs, polyunsaturated fatty acids. Highly abundant in many mammalian milks. Some species produce only one isoform, and others, such as humans, produce none.

*This identification from BLAST searching of GenBank with nearest similarity in *Canis lupus familiaris* database, but also *Ursus maritimus*; the *A. melanoleuca* NCBI GenBank GeneInfo accession code given here.

**Good identification as an immunoglobulin. Low level but positive identification as containing immunoglobulin μ chain, but migration rate in protein electrophoresis consistent with IgM.

*** One of the pandas (Xiao Yatou) produced a higher molecular sized isoform of this enzyme, possibly reflecting a polymorphism in glycosylation of this enzyme within the species (not shown). Such glycosylation could be relevant to protection against pathogens (ref. (1) and see also below).

For this table, all known identifications of trypsin (autoproteolysis during trypsin digestion stage) and keratin peaks (from panda or human skin and hair) were excluded.

Table S4. Relative abundance of the 10 most common small molecules in giant panda milks in colostrum and mature milk, compared with mature phase human and cow milks. Relative abundance is ranked according to peak heights and areas under the mass spectrometry data peaks. The giant panda samples were collected on the indicated days postpartum and the human and bovine samples were collected during mid lactation. Compounds listed in bold are discussed in the text. Time-dependent trends of fucosyllactose and lactose are presented in Figures 4 and S3, respectively. PC, phosphatidylcholine. PE, phosphatidylethanolamine.

**Giant panda day 3**	**Giant panda day 46**	**Giant panda day 158**	**Human**	**Cow**
Glycerophosphocholine	Glycerophosphocholine	Glycerophosphocholine	**Lactose**	Valine
**Sialyl lactose**	**Sialyl lactose**	**Fucosyllactose**	Choline	Creatine
**Lactose**	**Lactose**	Creatinine	Glutamine	acetylcarnitine
Creatine	Lyso PC 16:0	Carnitine	Alanine	**Lactose**
Lyso PC 16:0	Carnitine	Lyso PC 16:0	Acetylcarnitine	Creatinine
Creatinine	**Taurine**	Glycerophosphoethanolamine	Leucine	N-acetylglucosamine
Lyso PE 18:1	Lyso PE 18:1	Lyso PE 18:1	Glutamate	Carnitine
**Taurine**	Acetyl carnitine	**Taurine**	Glucose	riboflavin
Glycerophosphoethanolamine	Pentanoylcarnitine	Lyso PE 18:1	N-acetylglucosamine	N-acetylanthranilate
Lyso PC 18:0	Choline	Formiminotetrahydrofolate	Stachyose	Propionyl choline
N-acetylgalactosamine	Formiminotetrahydrofolate	Choline	Threonine	Octadecadienylglycerol

Table S5. Milk oligosaccharides reported in this and previous studies on giant pandas and other species of bear, and their post-partum trends in giant panda milk. This list is confined to those oligosaccharides definitively identified in this study – others were found but not fully identified. Their details are available on request. See also Figure S3 for graphical presentations of trends in concentrations with time after birth.

**Chemical formula**	**Subunit formula and name of compound**	**Reference**	**Observed post-partum trend in giant panda milk (this study)**
**Found in giant panda milk**			
C12H22O11	Lactose *	(2, 3)	Initially high, gradually decreases.
C23H39NO19	Neu5Ac(α2-3)Gal(β1-4)Glc (3’-N-acetylneuraminyllactose)	(2, 3)	Initially high, gradually decreases.
C23H39NO19	Neu5Ac(α2-3)Gal(β1-4)Glc (6’-N-acetylneuraminyllactose)	(2)	Initially low, gradual increase to plateau after 20 days.
C29H49NO23	Neu5Ac(α2-3)Gal(β1-4)Fuc(α1-3)Glc	(2)	Zero or low, rapid increase after 7 days to plateau after 20 days.
C18H32O16	Gal(α1-3)Gal(β1-4)Glc (isoglobotriose)	(2, 3)	Gradual increase to plateau after 20 days.
C24H42O20	Gal(α1-3)Gal(β1-4)Fuc(α1-3)Glc (fucosyl isoglobotriose)	(2, 3)	Initially zero or low then rapid increase after 7 days to plateau after 20 days.

* Lactose is the dominant sugar in cow and human milks, but is low or absent in the milks of many Carnivora.

Table S5 continued.

**Chemical formula**	**Subunit formula and name of compound**	**Reference**	**Observed post-partum trend in giant panda milk (this study)**
**Found in both giant panda milk and in the milks if other species of bear**			
C18H32O15	Fuc (α1-2)Gal(β1-4)Glc (2'-fucosyl lactose)	(3-6)	Initially low then dramatic increase after 20 days.

C26H45NO20	GalNAc(α1-3)[Fuc(α1-2)]Gal(β1-4)Glc (tetrasaccharide) (human blood group A determinant)	(3, 6)	Zero or low until 20 days then dramatic increase.
C62H102N4O47	Neu5Ac(α2-6)Gal(β1-4)GlcNAc(β1-3)[Neu5Ac(α2-6)Gal(β1-4)GlcNAc(β1-6)]Gal(β1-4)Glc	(7)	Initially low then increase after 20 days.
C38H65NO30	Gal(α1-3)Gal(β1-4)[Fuc(α1-3)]GlcNAc(β1-3)Gal(β1-4)Glc	(4, 6)	Low until 20 days then rapid increase.
C44H75NO34	Gal(α1-3)[Fuc(α1-2)]Gal(β1-4)[Fuc(α1-3)]GlcNAc(β1-3)Gal(β1-4)Glc (difucosylheptasaccharide)	(5)	Low until 20 days then rapid increase.
C63H105N3O48	Neu5Ac(α2-6)Gal(β1-4)GlcNAc(β1-3){Gal(α1-3)Gal(β1-4)[Fuc(α1-3)]GlcNAc(β1-6)}Gal(β1-4)Glc	(7)	Initially low then increases after 20 days.
C64H108N2O49	Gal(α1-3)Gal(β1-4)[Fuc(α1-3)]GlcNAc(β1-3)[Gal(α1-3)Gal(β1-4)[Fuc(α1-3)]GlcNAc(β1-6)]Gal(β1-4)Glc (difucosyldecasaccharide)	(5, 6)	Initially low then increases after 20 days.

Figure S3. Diversity in the concentration time courses of selected giant panda milk oligosaccharides with time after birth.

Time course plots of seven of the oligosaccharides listed in table S4 to illustrate the diversity in the timings of their appearances or reductions with time after birth. Particularly notable are those that are undetectable at first then rise in relative concentrations abruptly but at different times.

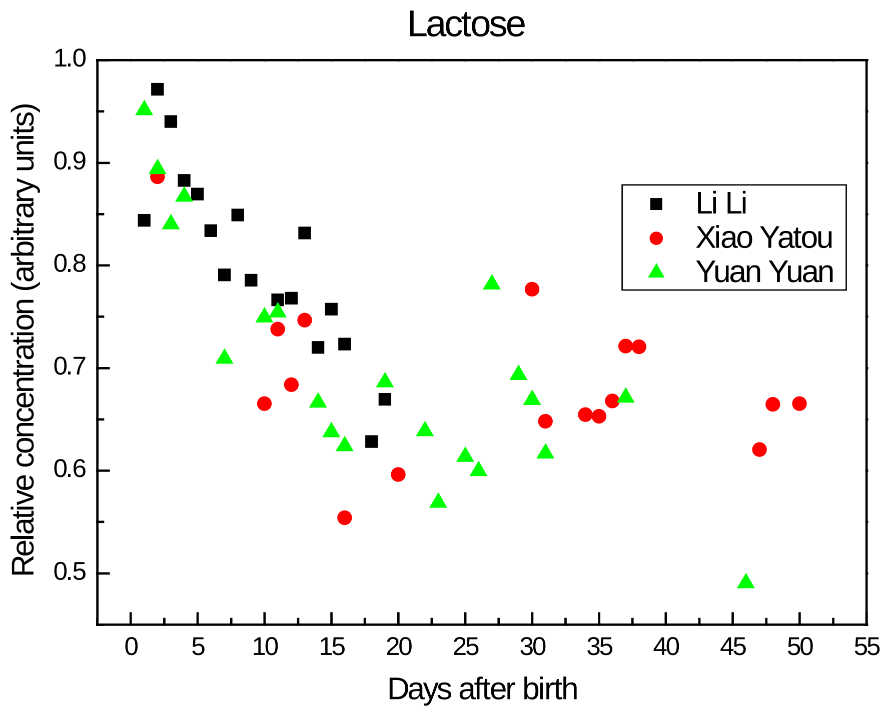

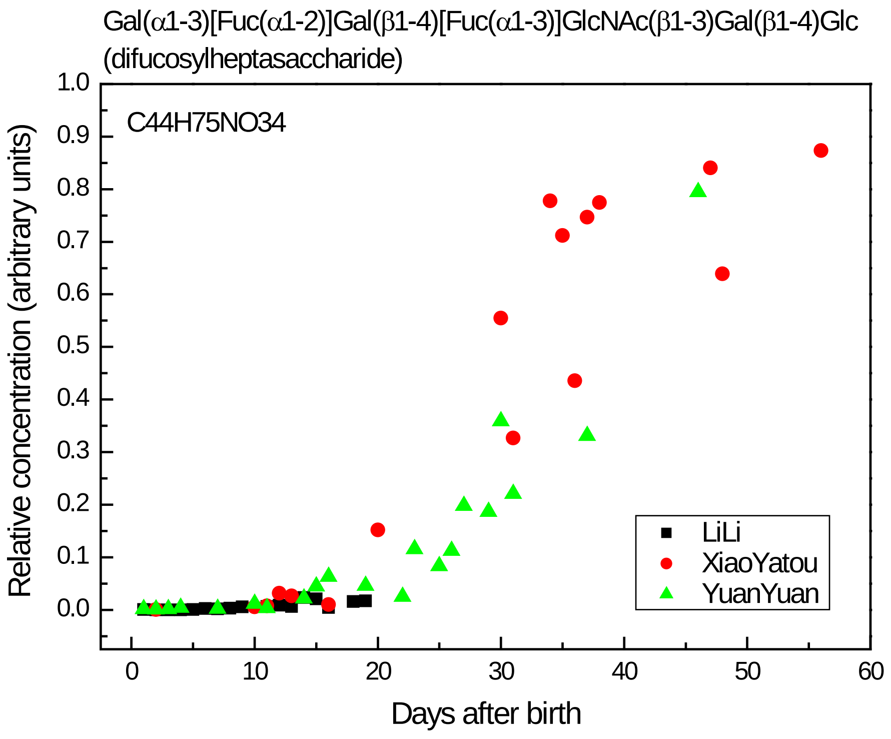

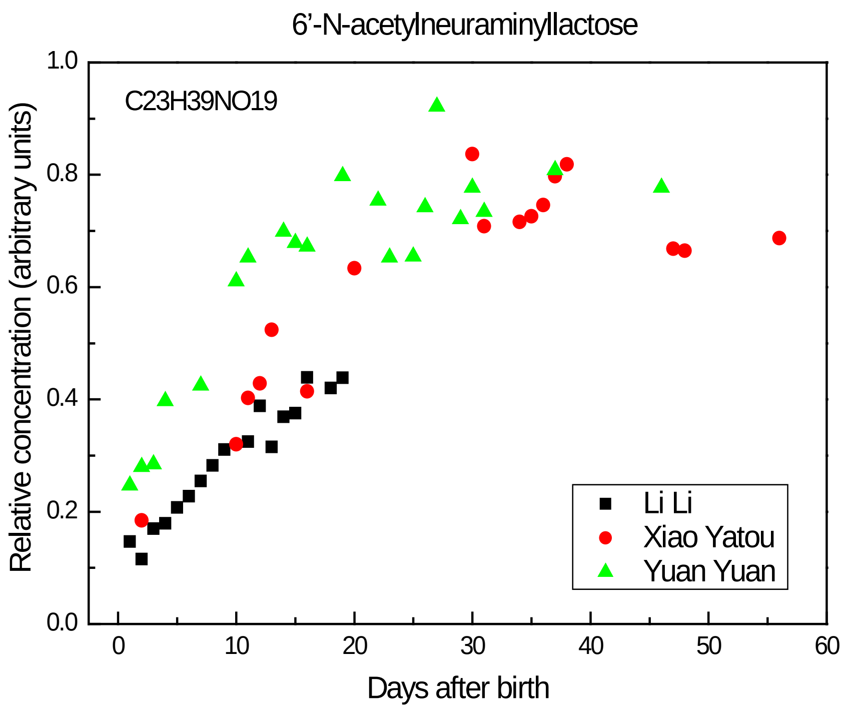
6’-N-acetylneuraminyllactose
C23H39NO19
Relative concentration (arbitrary units)
Days after birth
Li Li
Xiao Yatou
Yuan Yuan

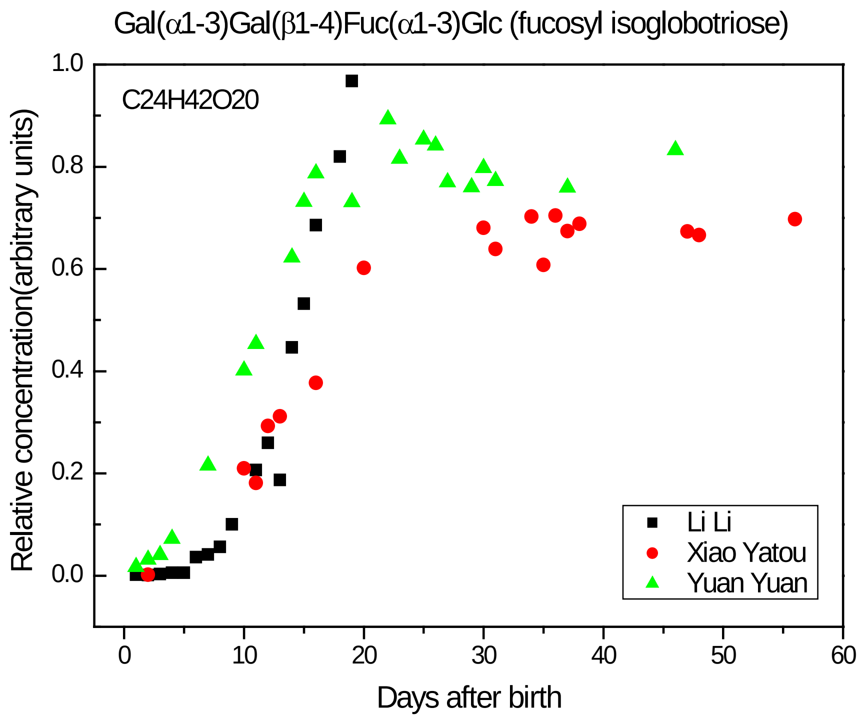
Gal(α1-3)Gal(β1-4)Fuc(α1-3)Glc (fucosyl isoglobotriose)
C24H42O20
Relative concentration(arbitrary units)
Days after birth
Li Li
Xiao Yatou
Yuan Yuan

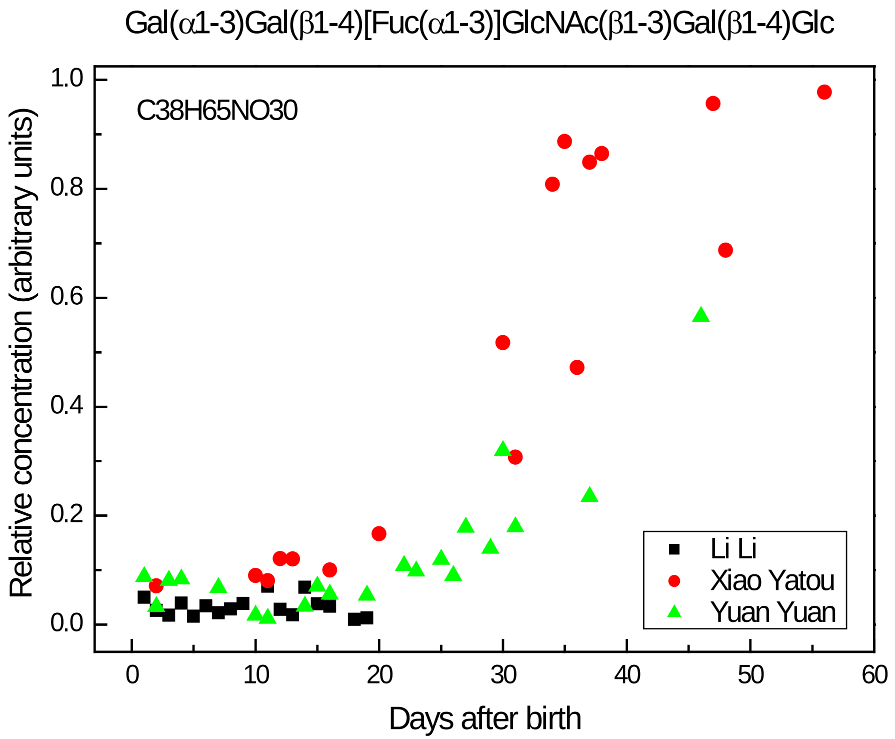
Gal(α1-3)Gal(β1-4)[Fuc(α1-3)]GlcNAc(β1-3)Gal(β1-4)Glc
C38H65NO30
Relative concentration (arbitrary units)
Days after birth
Li Li
Xiao Yatou
Yuan Yuan

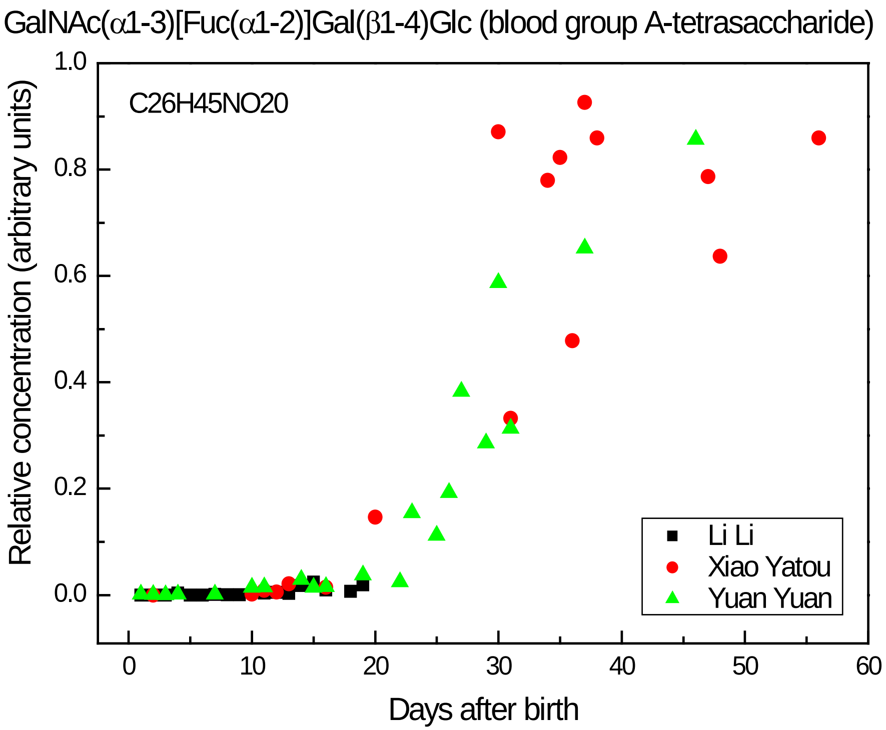
GalNAc(α1-3)[Fuc(α1-2)]Gal(β1-4)Glc (blood group A-tetrasaccharide)
C26H45NO20
Relative concentration (arbitrary units)
Days after birth
Li Li
Xiao Yatou
Yuan Yuan

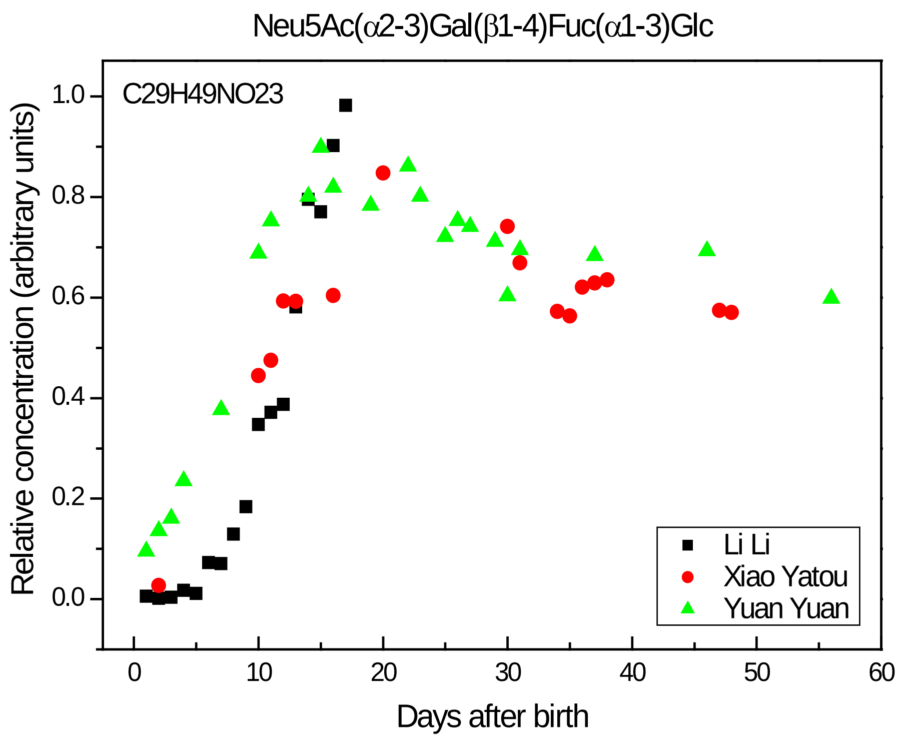
Neu5Ac(α2-3)Gal(β1-4)Fuc(α1-3)Glc
C29H49NO23
Relative concentration (arbitrary units)
Days after birth
Li Li
Xiao Yatou
Yuan Yuan

References for electronic supplementary material

1. Ruvoen-Clouet N, Mas E, Marionneau S, Guillon P, Lombardo D, Pendu JL. 2006 Bile-salt-stimulated lipase and mucins from milk of 'secretor' mothers inhibit the binding of Norwalk virus capsids to their carbohydrate ligands. *Biochemical Journal* **393,** 627-634. (doi: 10.1042/bj20050898)
2. Nakamura T, Urashima T, Mizukami T, Fukushima M, Arai I, Senshu T, Imazu K, Nakao T, Saito T, Ye ZY, Zuo H, Wu KJ. 2003 Composition and oligosaccharides of a milk sample of the giant panda, *Ailuropoda melanoleuca*. *Comparative Biochemistry and Physiology B-Biochemistry & Molecular Biology* **135,** 439-448. (doi: 10.1016/s1096-4959(03)00093-9)
3. Urashima T, Nagata H, Nakamura T, Arai I, Saito T, Imazu K, Hayashi T, Derocher AE, Wiig O. 2003 Differences in oligosaccharide pattern of a sample of polar bear colostrum and mid-lactation milk. *Comparative biochemistry and physiology Part B, Biochemistry & molecular biology* **136,** 887-896.
4. Urashima T, Kusaka Y, Nakamura T, Saito T, Maeda N, Messer M. 1997 Chemical characterization of milk oligosaccharides of the brown bear, Ursus arctos yesoensis. *Biochimica et biophysica acta* **1334,** 247-255.
5. Urashima T, Sumiyoshi W, Nakamura T, Arai I, Saito T, Komatsu T, Tsubota T. 1999 Chemical characterization of milk oligosaccharides of the Japanese black bear, Ursus thibetanus japonicus. *Biochimica et biophysica acta* **1472,** 290-306.
6. Urashima T, Yamashita T, Nakamura T, Arai I, Saito T, Derocher AE, Wiig O. 2000 Chemical characterization of milk oligosaccharides of the polar bear, Ursus maritimus. *Biochimica et biophysica acta* **1475,** 395-408.
7. Urashima T, Nakamura T, Teramoto K, Arai I, Saito T, Komatsu T, Tsubota T. 2004 Chemical characterization of sialyl oligosaccharides in milk of the Japanese black bear, Ursus thibetanus japonicus. *Comparative biochemistry and physiology Part B, Biochemistry & molecular biology* **139,** 587-595. (doi: 10.1016/j.cbpc.2004.07.012)
